# Integration of single-cell transcriptomics and bulk transcriptomics to explore prognostic and immunotherapeutic characteristics of nucleotide metabolism in lung adenocarcinoma

**DOI:** 10.3389/fgene.2024.1466249

**Published:** 2025-01-08

**Authors:** Kai Zhang, Luyao Wang, Huili Chen, Lili Deng, Mengling Hu, Ziqiang Wang, Yiluo Xie, Chaoqun Lian, Xiaojing Wang, Jing Zhang

**Affiliations:** ^1^ Anhui Province Key Laboratory of Respiratory Tumor and Infectious Disease, First Affiliated Hospital of Bengbu Medical University, Bengbu, China; ^2^ Department of Clinical Medicine, Bengbu Medical University, Bengbu, China; ^3^ Department of Genetics, School of Life Sciences, Bengbu Medical University, Bengbu, China; ^4^ Research Center of Clinical Laboratory Science, Bengbu Medical University, Bengbu, China; ^5^ Joint Research Center for Regional Diseases of IHM, The First Affiliated Hospital of Bengbu Medical University, Bengbu, China

**Keywords:** lung adenocarcinoma, nucleotide metabolism, single-cell transcriptome, prognostic model, immunotherapy

## Abstract

**Background:**

Lung adenocarcinoma (LUAD) is a highly aggressive tumor with one of the highest morbidity and mortality rates in the world. Nucleotide metabolic processes are critical for cancer development, progression, and alteration of the tumor microenvironment. However, the effect of nucleotide metabolism on LUAD remains to be thoroughly investigated.

**Methods:**

Transcriptomic and clinical data of LUAD were downloaded and organized from TCGA and GEO databases. Genes related to nucleotide metabolism were downloaded from the Msigdb database. Genes associated with LUAD prognosis were identified using univariate COX analysis, and a prognostic risk model was constructed using the machine learning combination of Lasso + Stepcox. The model’s predictive validity was evaluated using KM survival and timeROC curves. Based on the prognostic model, LUAD patients were classified into different nucleotide metabolism subtypes, and the differences between patients of different subtypes were explored in terms of genomic mutations, functional enrichment, tumor immune characteristics, and immunotherapy responses. Finally, the key gene SNRPA was screened, and a series of *in vitro* experiments were performed on LUAD cell lines to explore the role of SNRPA in LUAD.

**Result:**

LUAD patients could be accurately categorized into subtypes based on the nucleotide metabolism-related prognostic risk score (NMBRS). There were significant differences in prognosis between patients of different subtypes, and the NMBRS showed high accuracy in predicting the prognosis of LUAD patients. In addition, patients of different subtypes showed significant differences in genomic mutation and functional enrichment and exhibited different anti-tumor immune profiles. Importantly, NMBRS can be used to predict the responsiveness of LUAD patients to immunotherapy. The results of *in vitro* cellular experiments indicate that SNRPA plays an important role in the development and progression of lung adenocarcinoma.

**Conclusion:**

This study comprehensively reveals the prognostic value and clinical application of nucleotide metabolism in LUAD. A prognostic signature constructed based on genes related to nucleotide metabolism accurately predicted the prognosis of LUAD patients, and this signature can be used as a guide for LUAD immunotherapy.

## Introduction

Lung cancer has the highest morbidity and mortality rates in the world (Sung et al., 2021). About 85% of lung cancer cases are categorized as non-small cell lung cancer (NSCLC), and within this category, lung adenocarcinoma constitutes around 50% of the diagnoses (Sivakumar et al., 2017). LUAD is characterized by a high degree of malignancy, with the overall 5-year relative survival rate of patients being less than 20% (Siegel et al., 2020). Metabolic reprogramming is a mechanism by which cells alter their metabolic pathways to meet energy and material needs, promoting cell proliferation and growth. The reprogramming of tumor metabolism is essential for the continuation of tumor growth and viability, and it also affects the expression of immune-related molecules utilizing metabolite secretion., thereby regulating anti-tumor immune responses more broadly (Xia et al., 2021). In recent years, with the further study of tumor metabolic reprogramming and tumor immune microenvironment, tumor-targeted drugs and immunotherapy have been widely applied (Elmore et al., 2021). Despite these treatments’ efficacy, many LUAD patients still do not achieve effective treatment outcomes with current regimens (Brahmer et al., 2018). Hence, it is imperative to investigate novel LUAD biomarkers to enhance its prognostic evaluation and therapeutic strategies.

As key components in synthesizing intracellular DNA and RNA, nucleotides play a crucial role in cell growth, proliferation, and function (Rudolph, 1994). Unrestricted growth and proliferation are hallmark features of tumor cells, which depend on nucleotide metabolism to produce and maintain dNTP. Furthermore, the metabolism of nucleotides is intricately linked to the processes of DNA damage response and repair within cancer cells. By inhibiting pathways related to nucleotide metabolism, it is possible to specifically induce DNA damage in tumor cells (Helleday and Rudd, 2022). It is worth noting that nucleotide metabolism not only provides the necessary material basis for the growth and proliferation of tumor cells but also that related products of nucleotide metabolism in tumor cells can act as signaling molecules to convey information, and some substances can participate in the regulation of the tumor immune microenvironment (Ma et al., 2021). Therefore, studying nucleotide metabolism can help identify therapeutic targets for LUAD patients and provide new clinical diagnostic and treatment strategies.

This study aims to explore the prognostic and immunotherapeutic characteristics of nucleotide metabolism in LUAD to identify new therapeutic targets for lung adenocarcinoma. Initially, we examined the function of nucleotide metabolism in the development of LUAD by analyzing single-cell transcriptomic data. Then, based on the prognostic-related genes of nucleotide metabolism in LUAD, we constructed a prognostic model. The constructed prognostic feature can accurately stratify LAUD patients, and different subtypes of patients exhibit distinct prognoses, genomic mutations, functional enrichment, and tumor immune characteristics. Significantly, this prognostic characteristic has the potential to forecast the responsiveness of LAUD patients to immunotherapy treatments. In addition, by drug sensitivity correlation analysis we explored sensitive drugs for specific subtypes of LUAD patients. Finally, for the key gene SNRPA in nucleotide metabolism, we further identified SNRPA as a potential biomarker for LUAD by exploring its clinical prognostic value and preliminary experimental validation.

## Materials and methods

### Collecting and processing of bulk transcriptome data

We got gene sets associated with nucleotide metabolism (Supplementary Table S1) from the Msigdb database (https://www.gsea-msigdb.org/gsea/msigdb). Transcriptome and clinical data for LUAD patients were downloaded and organized from the TCGA database (https://portal.gdc.cancer.gov/) and the GEO database (https://www.ncbi.nlm.nih.gov/geo/). For the TCGA-LUAD data, we used TPM-type data for subsequent analysis. In the GEO dataset, we incorporated three cohorts: GSE31210 (Okayama et al., 2012), GSE50081 (Der et al., 2014), and GSE72094 (Schabath et al., 2016), each encompassing LUAD patients with comprehensive survival data. Additionally, we collected four immunotherapy cohorts, IMvigor210 (Balar et al., 2017), GSE78220 (Hugo et al., 2016), GSE135222 (Jung et al., 2019), and GSE100797 (Lauss et al., 2017), to evaluate the immunotherapy response in LUAD patients.

### Procurement and analysis of single-cell transcriptomic data

We acquired single-cell transcriptome samples of 6 LUAD patients from the research of Philip Bischoff and others (Bischoff et al., 2021) and processed and analyzed the single-cell data using the R package “Seurat” (version 4.40) (Stuart et al., 2019). To maintain high-quality data, we performed quality control, including filtering the data to include only genes expressed in a minimum of 3 cells, excluding cells that had less than 200 or more than 5,000 genes expressed, and excluding cells with more than 15% mitochondrial gene content. Initially, we applied the “NormalizeData” function to normalize the single-cell data, followed by identifying the top 2000 genes with the highest variability using the “FindVariableFeatures” function for further analysis. Subsequently, we analyzed the principal component with the “RunPCA” function. We clustered the single-cell data using the “FindNeighbors” and “FindClusters” functions and used the R package “SingleR” to assist in annotating different cell subpopulations (Aran et al., 2019). Finally, we used tSNE to present the annotation results visually.

### Single-cell transcriptome analysis of nucleotide metabolism

We use the “AddModuleScore” function to assess the nucleotide metabolism levels of different cell types. Then, we used the R package “CopyKAT” to deduce malignant cell types among epithelial cells (Gao et al., 2021) and the R package “Aucell” to evaluate the nucleotide metabolism levels of malignant and normal cells (Aibar et al., 2017).

### Establishment and confirmation of prognostic models

We employed a univariate Cox regression analysis to identify nucleotide metabolism-related genes linked to the prognosis of LUAD patients, with a significance threshold set at P < 0.05 (van Dijk et al., 2008). Then, in the TCGA cohort, we further screened genes using a combination of Lasso and Stepcox regression analyses (Tibshirani, 1997) and constructed the nucleotide metabolism-related prognostic signature NMBRS. Within the GEO cohort, we confirmed the predictive accuracy of NMBRS for the prognostication of LUAD patients by conducting survival and timeROC analyses.

### Model comparison and clinicopathological analysis

We compiled 11 prognostic features related to LUAD from various studies and evaluated their accuracy by comparing their C-indexes. Then, we explored the association between NMBRS and various clinicopathological factors and examined its predictive effectiveness in patients with different pathological characteristics through survival analysis.

### Autonomous prognostic evaluation and development of a nomogram

In the OS, DSS, and PFI data of LUAD patients (Liu et al., 2018), we conducted univariate and multivariate COX analyses on NMBRS and related clinicopathological factors. Subsequently, to enhance the feature’s clinical application potential, we developed a nomogram model incorporating NMBRS to measure the anticipated survival probability for LUAD patients. To confirm the nomogram model’s precision, we used calibration curves, timeROC analysis, and decision curve analysis (DCA) to assess its practical utility in a clinical setting.

### Analysis of tumor mutations

Mutation data for LUAD patients were retrieved and compiled from the TCGA database. Using the R package “maftools,” we plotted mutation waterfalls for patients of different subtypes (Mayakonda et al., 2018). Next, we analyzed NMBRS, TMB, and MATH correlations and conducted a deeper analysis of SNV and CNV mutations in model genes. Additionally, we have analyzed the interactions involving the top 20 mutated genes across various patient subtypes and the pathways they regulate.

### Analysis of functional enrichment

Initially, we performed GSEA (Gene Set Enrichment Analysis) on patients belonging to various subtypes (Mootha et al., 2003; Subramanian et al., 2005). Subsequently, utilizing the HALLMARK gene sets, we conducted GSVA (Gene Set Variation Analysis) enrichment analysis on those patients and an in-depth study of the correlation between NMBRS and HALLMARK pathways (Hänzelmann et al., 2013). Additionally, we applied the R package “clusterProfiler” to conduct GO and KEGG enrichment analyses on the genes that exhibited differential expression across various patient subtypes (Wu et al., 2021), aiming to explore the gene pathways they are involved in.

### Analysis of the TME and immune cell infiltration

We used the R package “ESTIMATE” to evaluate and compare the differences among patients with different subtypes regarding Immunescore, Stromscore, and TumorPurity (Yoshihara et al., 2013). Next, we utilized the CIBERSORT and ssGSEA algorithms to examine the infiltration of immune cells in patients across various subtypes (Newman et al., 2015). Antigen presentation and immune checkpoints play crucial roles in anti-tumor immunity. Therefore, we also investigated the variations in the expression of genes involved in antigen presentation and immune checkpoint among patients with varying subtypes. To comprehend the immune distinctions between the two subtypes more profoundly, we assessed the distribution of patients across various subtypes within the TME classification (Bagaev et al., 2021). We obtained the immune seven-step cycle activity scores of LUAD patients from the TIP website (http://biocc.hrbmu.edu.cn/TIP/analysis.jsp) (Xu et al., 2018). Furthermore, we examined the variations in 13 immune functions across patients of different subtypes and explored the association between NMBRS and immune cell populations.

### Immunotherapy and drug sensitivity analysis

We retrieved TIDE scores for LUAD patients from the TIDE website (http://tide.dfci.harvard.edu) (Jiang et al., 2018) and IPS scores for LUAD patients from the TCIA website (https://www.tcia.at/home). Using this data, we compared the differences in TIDE, T-cell Exclusion, and IPS scores among different subtypes of patients. Submap is an unsupervised method that can estimate the association between subclasses observed in two independent datasets (Hoshida et al., 2007). We used the Submap algorithm to assess the responsiveness of various patient subtypes to immunotherapy treatments. A smaller P-value indicates a higher similarity between the two modules. Next, to further study the response of different subtypes of patients to immunotherapy, we assembled four different immunotherapy cohorts: IMvigor210, GSE78220, GSE135222, and GSE100797. We compared the immunotherapy response of different subtypes of patients across these different immunotherapy cohorts. The R package “oncoPredict” can assess patients’ sensitivity to standard chemotherapy drugs (Maeser et al., 2021). Our comparisons and screenings have identified chemotherapy drugs suitable for patients of different subtypes.

### Single-cell transcriptome analysis of NMBRS

We categorized epithelial cells into High_NMBRS_Epithelial_cells and Low_NMBRS_Epithelial_cells based on the expression of seven model genes in the epithelial cells. Using the “FindMarkers” function, we conducted a differential analysis of the different types of epithelial cells and further explored the related pathways involving the differential genes. Subsequently, we utilized the R package “cellchat” to investigate the cell communication relationships between different types of epithelial cells and other cell types within the TME (Jin et al., 2021).

### Screening of key genes and pan-cancer analysis

We used differential analysis, survival analysis, and ROC diagnostic curves to identify the nucleotide metabolism gene SNRPA, which is markedly linked to the prognostic outcomes of LUAD patients. Subsequently, we evaluated the expression levels of SNRPA across the TCGA pan-cancer dataset and conducted a univariate Cox regression analysis within the pan-cancer cohort. Additionally, we investigated the association of SNRPA with clinicopathologic factors in patients with LUAD.

### Spatial transcriptome RNA sequencing analysis of SNRPA

We obtained spatial transcriptomic data of 6 cases of lung adenocarcinoma from Zhu et al. (2022). Normalization, dimensionality reduction, clustering, and spatial variable characterization were performed using the R package “Seurat”. According to Zhu et al., pathologic HE-stained sections were divided into four regions: Cancer, Normal epithelium, Stromal, and Lymph. The “SpatialFeaturePlot” function was used to visualize the expression level and heterogeneity of SNRPA in different spatial locations.

### Cell culture and transfection

We performed *in vitro* culture experiments with A549 and H1299 cell lines, which were grown in DMEM and RPMI 1640 media (Gibco, ThermoFisher Scientific, United States), respectively, enriched with 10% fetal bovine serum, and 1% penicillin and streptomycin (Gibco). Small interfering RNA (siRNA) directed against SNRPA and control siRNA were obtained from Gima Genetics (Shanghai, China). To introduce these siRNAs transiently, A549 and H1299 cells were transfected for 24 h with siRNA using Lipofectamine 2000 transfection reagent, after which functional assays were carried out (Dalby et al., 2004).

### RT-qPCR experiment

RNA was isolated from lung adenocarcinoma cell lines (A549, H1299) and treated with si-SNRPA and negative control (NC) siRNA. Vazyme SYBR Green qPCR Master Mix (Vazyme, China) synthesized cDNA for real-time PCR analysis. The primer sequences are provided in Supplementary Table S2.

### Proliferation and colony formation assay

Cell proliferation and colony formation assays followed A549 and H1299 cell transfection with SNRPA siRNA for 24 h. The cells were plated at a density of 4000 cells per well in a 96-well plate. The growth capacity of the treated cells was evaluated at 24, 48, and 72 h using the Cell Counting Kit-8 (CCK8) reagent (Bio-sharp, Hefei, China), with OD450 measurements being taken using a microplate reader (BioTek, United States). In the colony formation assay, 1000 cells were seeded in culture dishes and allowed to grow until colonies became visible. The clones were then immobilized with paraformaldehyde for 15 min, colored with 1% crystal violet for 20 min, and numbers of colonies were counted.

### Transwell migration and invasion assay and wound healing assay

A549 and H1299 cells were subjected to Transwell migration and wound healing assays following 24 h of transfection with SNRPA siRNA. For the Transwell assay, cells were plated in 24-well plates with inserts with an 8 µm pore size to assess their migratory and invasive capabilities. Cells (4 × 10^4^) were placed in the upper compartment of the insert, which contained DMEM and RPMI 1640 without FBS, while the lower compartment was filled with 600 µL of DMEM and RPMI 1640 medium supplemented with FBS. After an incubation period of 48 h, the cells were fixed, stained with crystal violet, and enumerated under a light microscope at ×100 magnification. In addition, A549 and H1299 cells were seeded in 6-well plates, and a wound was created using a 200 µL pipette tip. The cells were then cultured in DMEM and RPMI 1640 media devoid of FBS. Wound images were taken at the start (0 h) and after 24 h, and the wound area was measured using ImageJ software.

### Data statistics

All statistical evaluations and graphical depictions were done using R-4.3.0 and GraphPad software. The Wilcoxon test was applied for two-group comparisons, and the t-test within GraphPad Prism determined the significance of cell line experiments. Survival analysis was performed using the Kaplan-Meier method with the “Survminer” package in R. Statistical significance was defined as P < 0.05.

## Results

### Nucleotide metabolic characteristics in single-cell transcriptome

**FIGURE 1:**
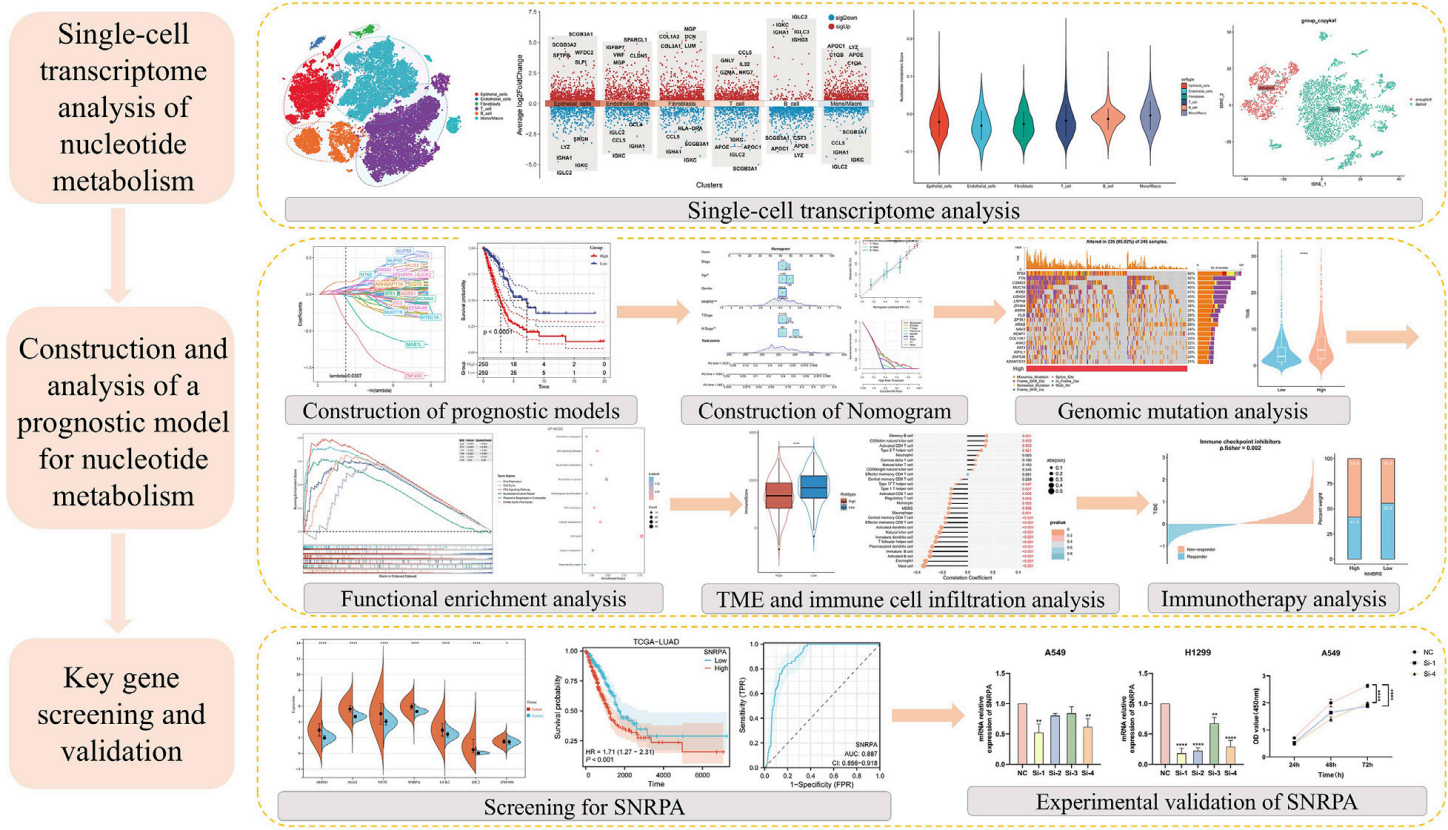
Flowchart of the study’s workflow.

**FIGURE 2:**
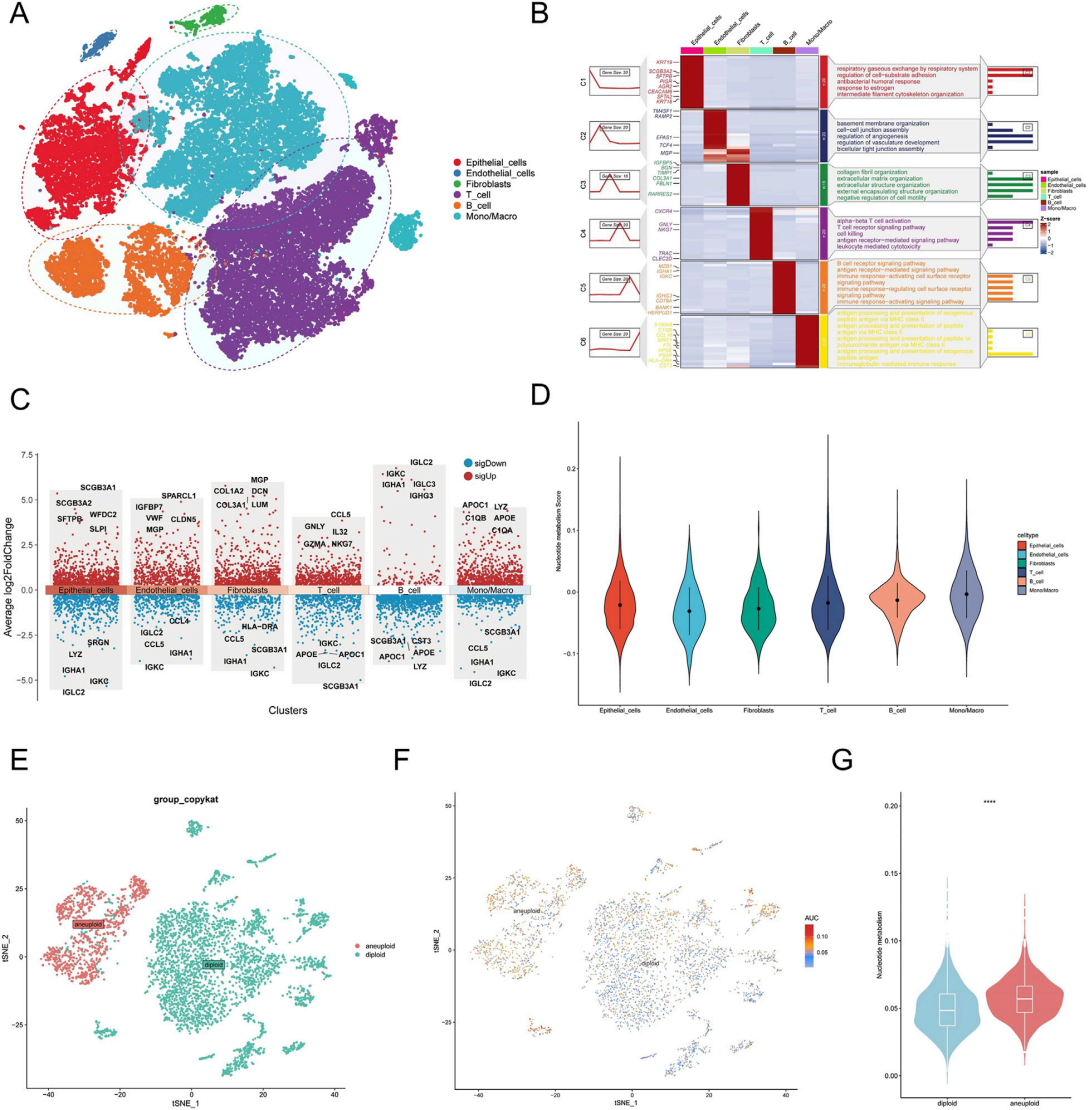
Single-cell transcriptome analysis of nucleotide metabolism. **(A)** Annotation results of single-cell subpopulations. **(B)** Heat map of marker genes in cellular subpopulations. **(C)** Volcano maps for differential analysis of subgroups. **(D)** Nucleotide metabolism levels in each cell type. **(E)** CopyKAT inference results of malignant cells in epithelial cells. **(F)** Distribution of nucleotide metabolism levels in normal and malignant cells. **(G)** Differences between the levels of nucleotide metabolism in normal and malignant cells. (*P < 0.05; **P < 0.01; ***P < 0.001; ****P < 0.0001).

Figure 1 depicts the entire workflow of this research. We obtained single-cell transcriptome samples from 6 cases of lung adenocarcinoma, resulting in 36,269 cells after quality control. Next, we studied the top 2000 variable genes using Principal Component Analysis and performed clustering analysis, grouping all cells into 26 clusters. With the help of singleR, we classified the cells into six different cell types, including Epithelial_cells, Endothelial_cells, Fibroblasts, T_cell, B_cell, and Mono/Macro. We used the “tSNE’ method to visualize the annotation results of these cells (Figure 2A). The heatmap and volcano plot, respectively, display the marker genes in different types of cells and the significantly upregulated and downregulated genes (Figures 2B, C). To accurately quantify the levels of nucleotide metabolism in other cell types, we employed the “AddModuleScore” function to compute the expression levels of genes associated with nucleotide metabolism across all cells within six distinct cell types, and we noticed significantly higher levels of nucleotide metabolism in Epithelial_cells, T_cell, B_cell, and Mono/Macro cells (Figure 2D). Then, we used the CopyKAT package to infer the type of malignant cells in epithelial cells (Figure 2E) and utilized the Aucell package to assess nucleotide metabolism in malignant and normal cells. We observed a significant increase in nucleotide metabolism in malignant cells (Figures 2F, G). This suggests that nucleotide metabolism is essential in the development of LUAD.

### Construction and validation of risk characteristics in nucleotide metabolism

**FIGURE 3:**
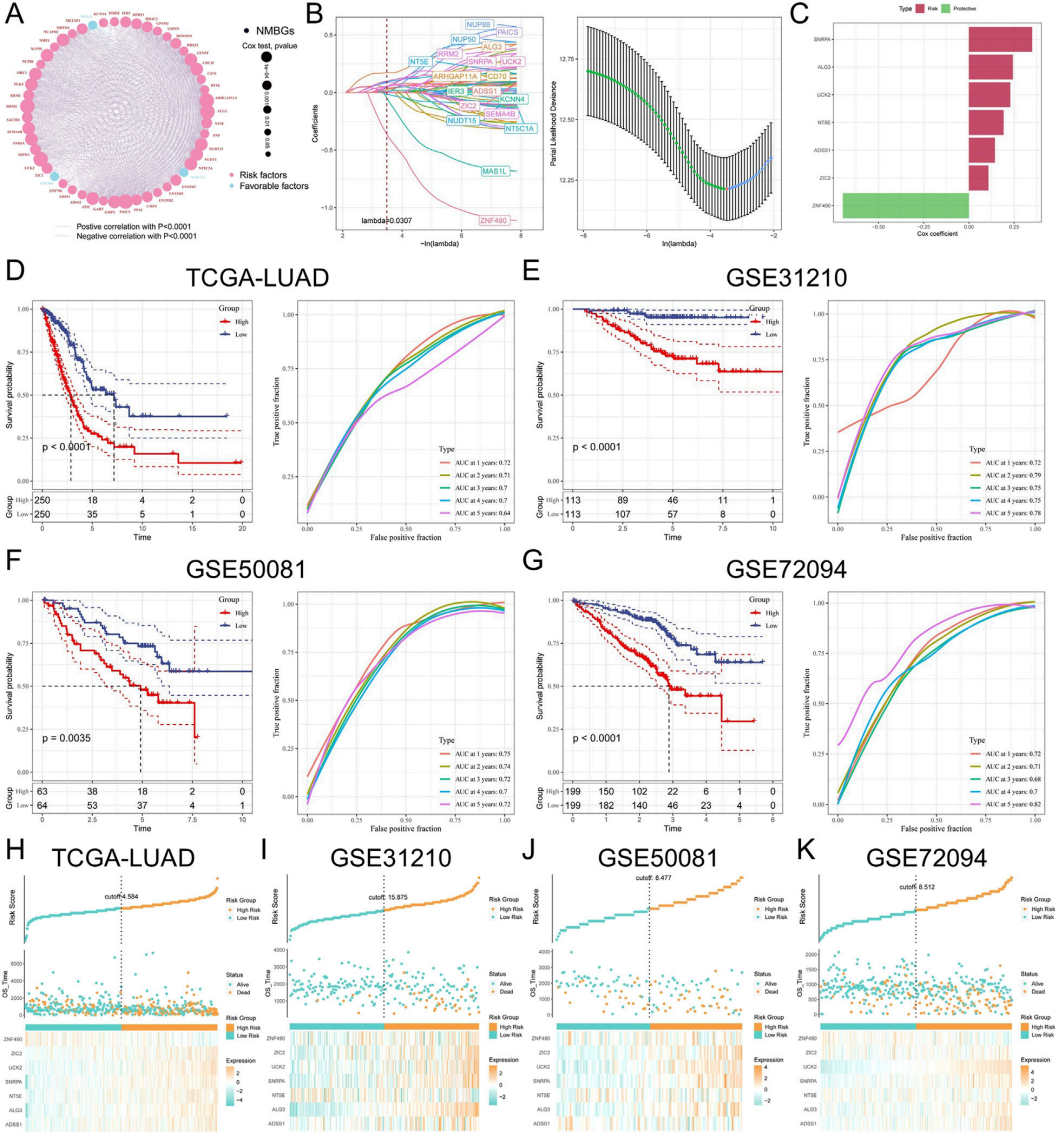
Modeling prognosis related to nucleotide metabolism. **(A)** Prognosis-related nucleotide metabolism genes and their interactions in LUAD. **(B)** Lasso regression analysis and cross-validation. **(C)** Seven genes were selected for modeling through Stepcox regression analysis. **(D)** KM curve and timeROC curve for the TCGA-LUAD queue. **(E)** KM curve and timeROC curve for the GSE31210 queue. **(F)** KM curve and timeROC curve for the GSE50081 queue. **(G)** KM curve and timeROC curve for the GSE72094 queue. **(H–K)** Distribution of OS status, OS, risk scores in the TCGA and GEO cohorts, and heatmap of mRNA expression of the seven genes between the high NMBRS and low NMBRS groups.

To delve deeper into the correlation between nucleotide metabolism and the prognostic outcomes of LUAD patients, we utilized the TCGA-LUAD dataset as a training dataset to build the model. Initially, we conducted a univariate Cox regression analysis to identify nucleotide metabolism genes associated with the prognosis of LUAD patients (Figure 3A). Subsequently, we utilized Lasso regression analyses to decrease the number of variables (Figure 3B). We employed Stepcox regression analysis to reduce the number of variables further and obtain the modeling genes’ risk coefficients (Figure 3C). Based on gene expression levels and risk coefficients, we derived the prognostic feature NMBRS for LUAD nucleotide metabolism, with the calculation formula as NMBRS = SNRPA*0.35 + ALG3*0.243 + UCK2*0.229 + NT5E*0.192 + ADSS1*0.144 + ZIC2*0.108 + ZNF490*-0.697. We divided LUAD patients into groups with high and low NMBRS scores, using the median NMBRS value as the cutoff. The survival analysis showed that patients with high NMBRS had a significantly worse prognosis (P < 0.0001). The timeROC curves showed that the AUC values for NMBRS from 1 to 5 years were 0.72, 0.71, 0.70, 0.70, and 0.64, respectively (Figure 3D). The prognosis of patients with a high subgroup of NMBRS was similarly worse in the GEO validation set, with the AUC values from 1 to 5 years exceeding 0.68 (Figures 3E–G). These results suggest that NMBRS has high accuracy in predicting prognosis in patients with LUAD. In addition, patients in the low NMBRS group in the TCGA and GEO cohorts had significantly longer survival (Figures 3H–K).

### Model comparison and clinical pathological analysis of NMBRS

**FIGURE 4:**
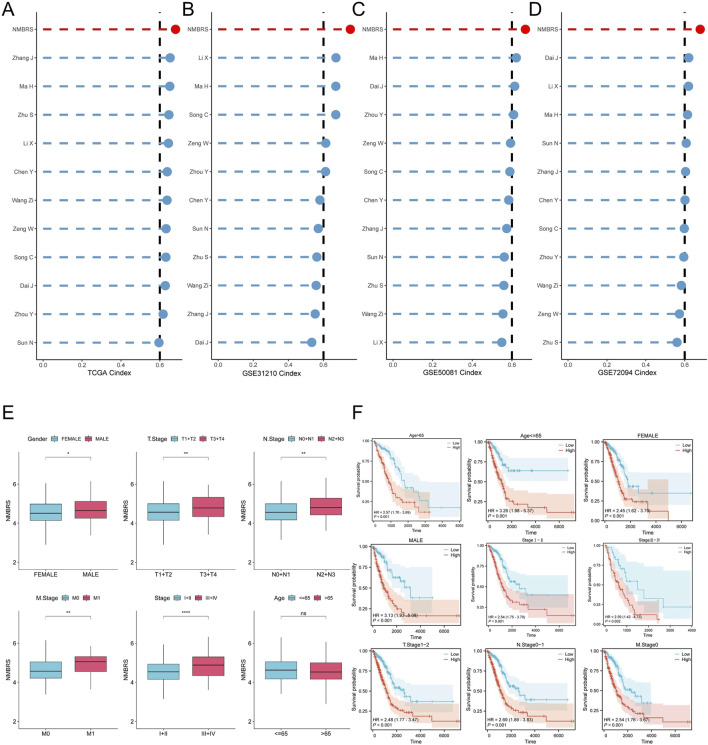
Model comparison and clinicopathological analysis. **(A–D)** NMBRS compared with the C index of 11 LUAD-related studies in the TCGA-LUAD cohorts, the GSE31210 cohorts, the GSE50081 cohorts, and the GSE72094 cohorts. **(E)** Correlation between NMBRS and various clinicopathological factors. **(F)** Predictive performance of NMBRS across different clinicopathological factors. (*P < 0.05; **P < 0.01; ***P < 0.001; ****P < 0.0001).

We collected models from 11 LUAD-related studies and compared the C-index of these models. The results showed that the C-index of NMBRS was higher than these models in the TCGA and GEO cohorts (Figures 4A–D), indicating that NMBRS has higher accuracy and superiority than these models. Next, we analyzed the relationship between NMBRS and various clinicopathological factors, and the findings indicated higher NMBRS scores in male patients and patients with advanced chronologic late TNM staging (Figure 4E). Patients with high NMBRS also had significantly poorer prognoses across different clinicopathological characteristics (Figure 4F).

### Independent prognostic analysis and development of a nomogram

To establish if NMBRS serves as an independent prognostic indicator for LUAD patients, we conducted both univariate and multivariate Cox regression analyses on NMBRS alongside a range of clinicopathological characteristics within the TCGA-LUAD dataset, focusing on OS, DSS, and PFI. The findings suggest that NMBRS acts as an independent risk factor affecting the prognosis of LUAD patients, as evidenced by both univariate and multivariate Cox regression analyses (HR > 1 and P < 0.001) (Supplementary Figures S1A, B). Furthermore, we noted that for LUAD patients in terms of DSS and PFI, those in the high NMBRS group had a significantly poorer prognosis than those in the low NMBRS group (Supplementary Figures S1C, D). Subsequently, to improve the clinical applicability of NMBRS, we developed a nomogram that incorporated NMBRS and a range of clinicopathological variables (Supplementary Figure S1E). The calibration curve analysis revealed that the nomogram’s predictions were well-aligned with actual observations (Supplementary Figure S1F). The timeROC analysis showed that the AUC values of the nomogram and NMBRS were markedly higher than those of other clinicopathological characteristics (Supplementary Figure S1G). Decision curve analysis (DCA) revealed that the nomogram and NMBRS offered superior net clinical benefits compared to other clinicopathological characteristics (Supplementary Figure S1H). PCA analysis demonstrated that NMBRS could accurately classify LUAD patients into two groups (Supplementary Figure S1I).

### Genomic mutation characteristics of different subtypes of patients

**FIGURE 5:**
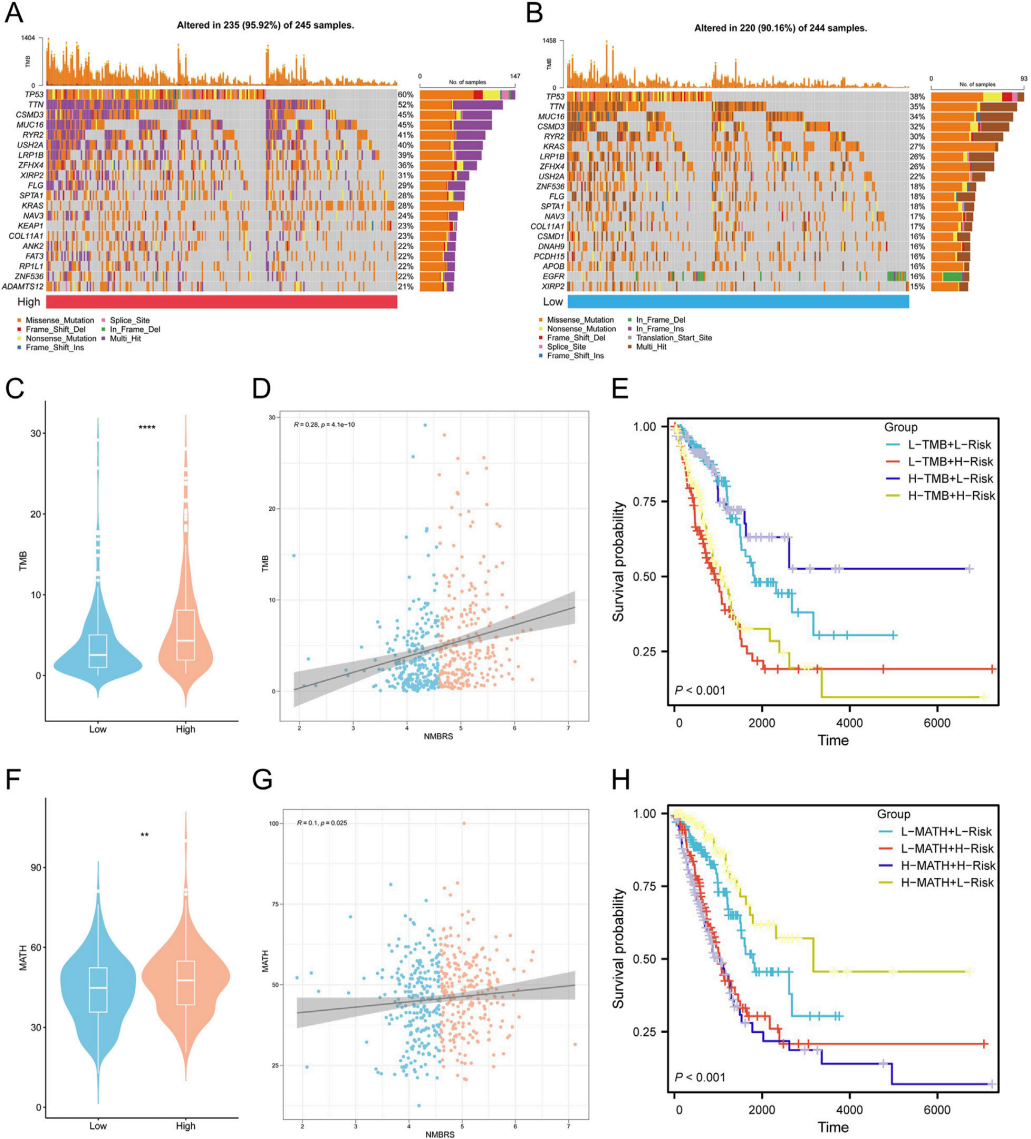
NMBRS-related genetic alterations between the low and high NMBRS groups. **(A)** A waterfall map of the mutation status of somatic cells in the high-NMBRS group. **(B)** A waterfall map of the mutation status of somatic cells in the low-NMBRS group. **(C)** The Violin plot demonstrates differences in TMB scores for the high NMBRS and low NMBRS groups. **(D)** The correlation of NMBRS with TMB. **(E)** Combined TMB score and NMBRS risk score for Kaplan-Meier curve analysis of OS. **(F)** The Violin plot demonstrates differences in MATH scores for the high NMBRS and low NMBRS groups. **(G)** The correlation of NMBRS with MATH. **(H)** Combined MATH score and NMBRS risk score for Kaplan-Meier curve analysis of OS. (*P < 0.05; **P < 0.01; ***P < 0.001; ****P < 0.0001).

Earlier research has demonstrated that somatic mutations are intricately linked to the development and progression of tumors (Martincorena and Campbell, 2015). Hence, we undertook to perform an in-depth investigation into the genomic mutation disparities between patients with high and low NMBRS scores and created mutation waterfall plots (Figures 5A, B). The findings indicated that the genomic mutation frequency was notably higher in patients with high NMBRS scores, reaching 95.92%. In contrast, the genomic mutation frequency was relatively lower in patients in the low NMBRS group at 90.16%. Furthermore, we noted a substantial rise in TMB in the high NMBRS group and a positive correlation between NMBRS and TMB (Figures 5C, D). Patients with lower TMB and higher NMBRS had poorer prognosis (Figure 5E). Similarly, we detected a notable upsurge in MATH in the high NMBRS group, and there was a positive association between NMBRS and MATH (Figures 5F, G), with poorer prognosis in patients with higher MATH and high NMBRS (Figure 5H). Subsequently, we performed an in-depth analysis of the mutation status for the seven genes used in the model construction. Significant SNV mutations were observed in UCK2 and ALG3 (Supplementary Figure S2A), while high-frequency CNV amplifications were seen in UCK2, ALG3, SNRPA, and ZIC2, and notable CNV deletions were found in ADSS1, NT5E, and ZNF490 (Supplementary Figure S2B). We also illustrated the chromosomal locations of the seven genes (Supplementary Figure S2C). Furthermore, we conducted a correlation analysis of the top twenty mutated genes between the high and low NMBRS groups (Supplementary Figures S2D, E) and examined the impact of these mutated genes on cancer-related pathways (Supplementary Figures S2F, G). The findings suggested that in the high NMBRS group, the proportion of cancer pathways impacted by mutated genes was more pronounced. These analysis results indicated that patients with high NMBRS scores exhibit poorer genomic stability and a higher mutation rate compared to those with low NMBRS scores. This could be a crucial factor contributing to the worse prognosis observed in patients with high NMBRS scores.

### Potential molecular mechanisms by which NMBRS exerts its effects

**FIGURE 6:**
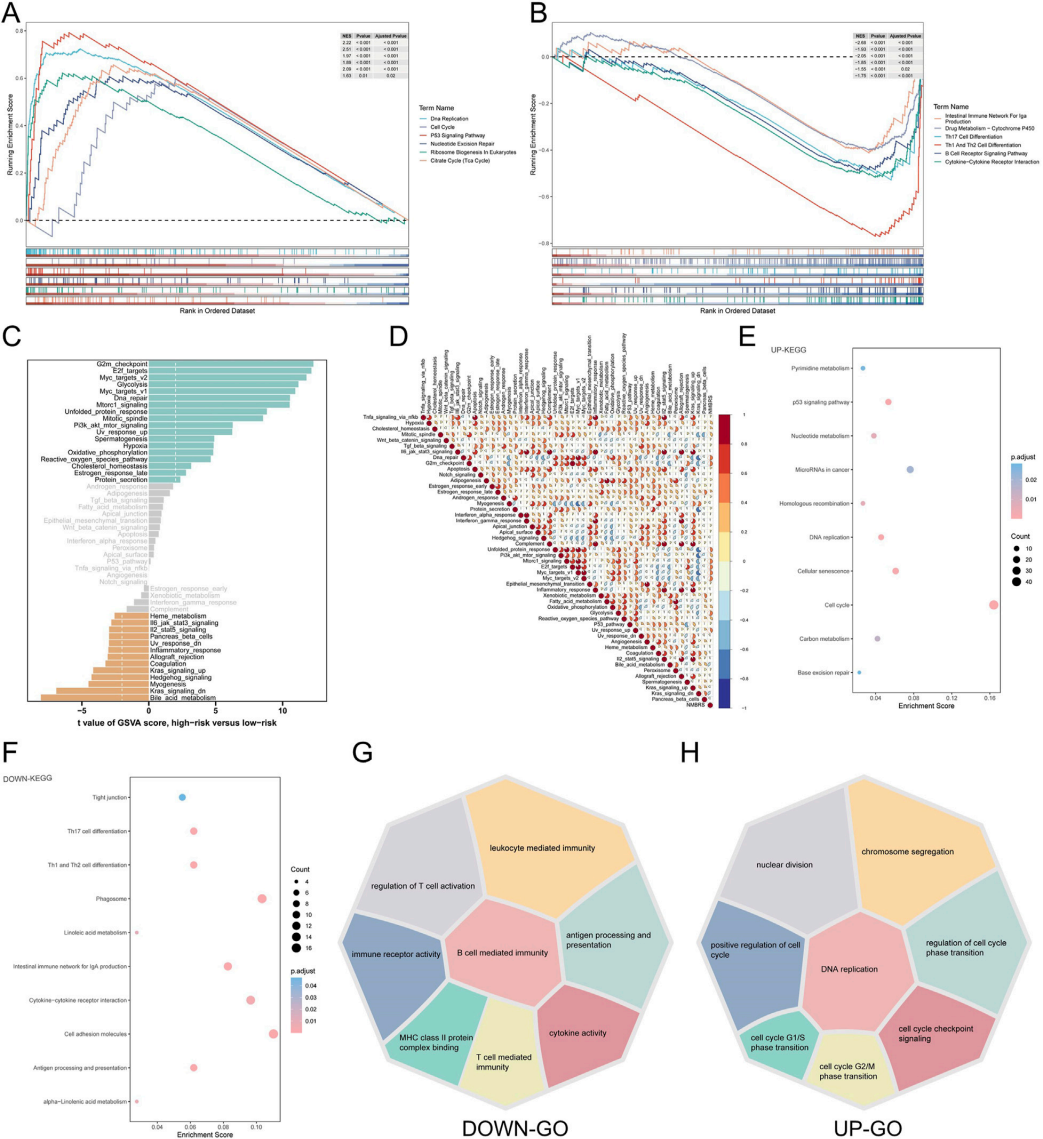
Functionally rich features associated with NMBRS. **(A)** Analysis of GSEA enrichment in patients with high-NMBRS group. **(B)** Analysis of GSEA enrichment in patients with low-NMBRS group. **(C)** The difference in the level of HALLMARK pathway activation according to GSVA scores between the high and low NMBRS groups. **(D)** The correlation of NMBRS with the HALLMARK pathway. **(E, F)** Analysis of KEGG enrichment for different genes in patients with various subtypes. **(G, H)** Analysis of GO enrichment for other genes in patients with other subtypes.

First, we performed GSEA enrichment analysis on patients in the high NMBRS and low NMBRS groups. The results showed that many cancer-related pathways were significantly activated in patients in the high NMBRS group. In contrast, many immune-related pathways were activated considerably in patients in the low NMBRS group (Figures 6A, B). We also observed significant activation of nucleotide metabolism pathways in patients in the high NMBRS group (Supplementary Figures S3A–C). Subsequently, we conducted GSVA enrichment analysis using the HALLMARK gene set on patients across various subtypes. The outcome indicates that patients with high NMBRS scores significantly activated cell cycle-related pathways, such as G2m_checkpoint and E2f_targets, and cell proliferation-related pathways, such as Pi3k_akt_mtor_signaling. Conversely, in the low NMBRS group, immune-related pathways, such as Inflammatory_response and Il6_jak_stat3_signaling, were significantly activated (Figure 6C). Additionally, we observed a significant positive correlation between NMBRS and pathways like Dna_repair, G2m_checkpoint, Pi3k_akt_mtor_signaling, and Myc_targets_v2, while a significant negative correlation was found with Bile_acid_metabolism and Kras_signaling_up (Figure 6D). We also performed GO and KEGG enrichment studies on the differentially expressed genes across various patient subtypes (Figures 6E–H). After analysis, we found that in patients with high NMBRS, the upregulated genes are mainly concentrated in cell cycle and proliferation-related pathways and processes. In contrast, in the low NMBRS group, the upregulated genes are primarily focused on immune function-related processes and pathways. Additionally, we utilized the PROGENy algorithm to gauge the activation status of tumor signaling pathways across diverse patient subtypes (Schubert et al., 2018). The study found that in the high NMBRS group, the activity of pathways such as EGFR, Hypoxia, MAPK, PI3K, VEGF, and WNT was significantly increased (Supplementary Figure S3D).

### Tumor immune characteristics of different subtypes of patients

**FIGURE 7:**
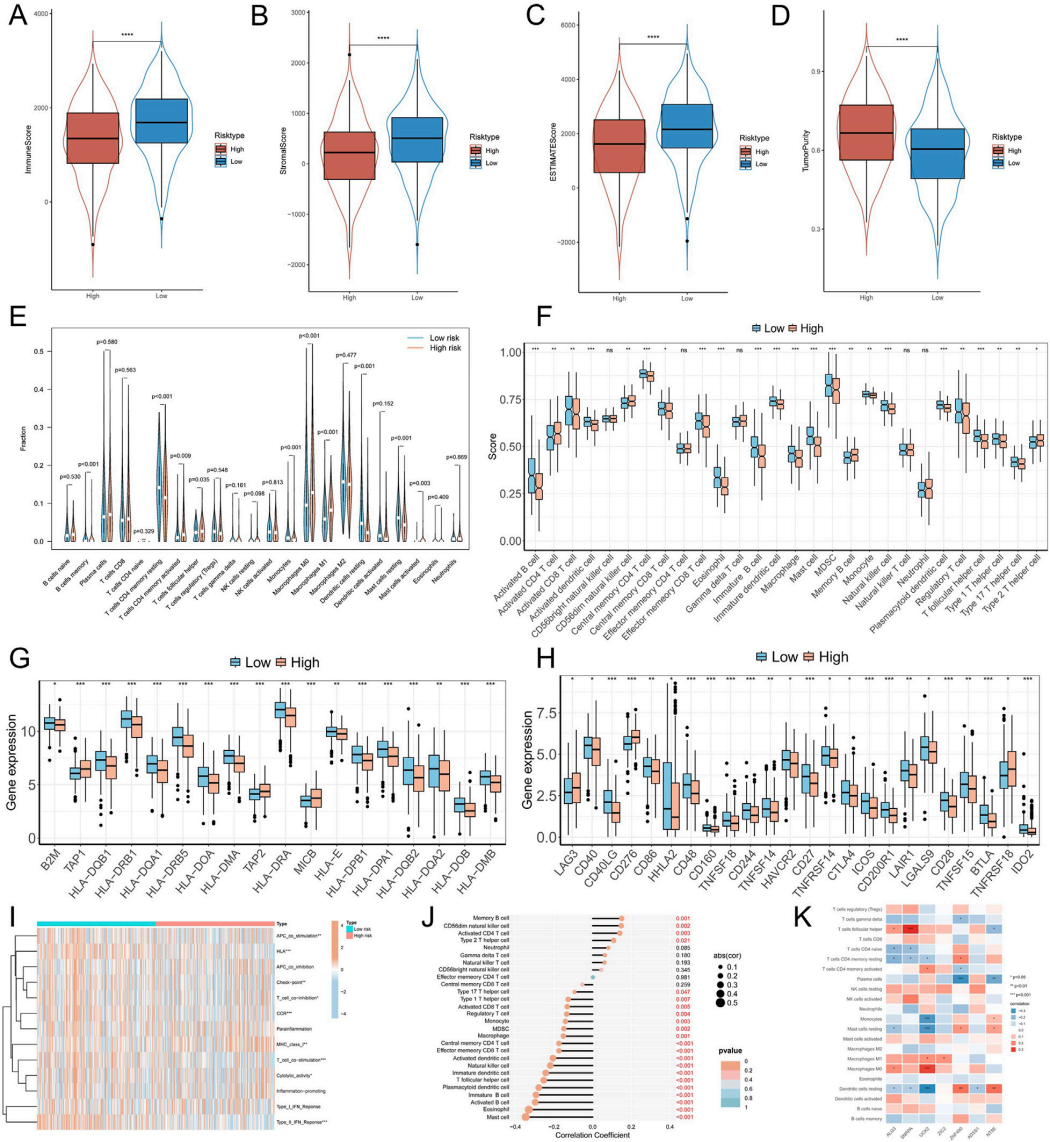
Immune landscape associated with NMBRS in LUAD. **(A–D)** Differences in Immunescore, Stromalscore, Tumorpurity, and ESTIMATEscore among patients with different subtypes. **(E)** The CIBESORT algorithm between the high NMBRS and low NMBRS groups calculated the abundance of various types with infiltrating cells. **(F)** Abundance levels for various immune-infiltrating cells were calculated using the ssGSEA algorithm among the high and low NMBRS groups. **(G, H)** Differential expression in antigen-presenting genes in patients with different subtypes and immune checkpoint genes. **(I)** Variation in activation level of 13 immunologic functions across subtypes of patients. **(J)** The correlation of immune-infiltrating cells with NMBRS. **(K)** Immune-infiltrating cells correlate with seven genes in NMBRS. (*P < 0.05; **P < 0.01; ***P < 0.001; ****P < 0.0001).

TME plays a significant role in antitumor immunity and tumor cell immune evasion. To investigate the immune features of patients with various subtypes, we utilized the ESTIMATE algorithm to calculate and contrast the variations in Immunescore, Stromascore, and TumorPurity among patients across different subtypes. We discovered that patients with low NMBRS scores exhibited higher Immunescore, Stromascore, and ESTIMATE scores (Figures 7A–C), while patients in the high NMBRS group displayed significantly higher TumorPurity (Figure 7D). Subsequently, we utilized the CIBERSORT and ssGSEA algorithms to evaluate and contrast the infiltration of immune cells among patients across different subtypes. The results from the CIBERSORT algorithm suggested that patients with low NMBRS scores had relatively higher levels of infiltration by Monocytes, resting Dendritic cells, and resting CD4 memory T cells (Figure 7E). The results of the ssGSEA algorithm suggest that patients with low NMBRS scores exhibit higher infiltration levels of Activated B cells, Activated CD8 T cells, Activated dendritic cells, and Monocytes (Figure 7F), which play crucial roles in anti-tumor immunity. Antigen presentation is a key step in anti-tumor immunity (Jhunjhunwala et al., 2021). We studied the expression values of antigen presentation genes in patients across various subtypes. The findings indicated that most antigen presentation genes were notably upregulated in patients with low NMBRS scores (Figure 7G). This demonstrates that patients in the lower NMBRS group had a higher capacity for tumor antigen presentation. In tumor immunity and immunotherapy, immune checkpoint genes play a critical role. We also assessed the expression values of immune checkpoints in patients across various subtypes and found that, apart from LAG3, CD276, and TNFRSF18, which were significantly increased in the high NMBRS group, most immune checkpoint genes had higher expression in the low NMBRS group (Figure 7H). This suggests that patients with low NMBRS scores may respond more effectively to immunotherapy. Next, we performed a comparative analysis of TME subtypes in patients with high and low NMBRS. The outcomes revealed that patients with high NMBRS scores were predominantly of the D and F subtypes (67%), whereas those with low NMBRS scores were mainly of the IE and IE/F subtypes (53%) (Supplementary Figure S3E). Antitumor immunity is a complex process involving multiple steps, including seven main steps (Chen and Mellman, 2013). We evaluated patients’ activity levels across various subtypes at these seven stages. It was discovered that patients with low NMBRS scores exhibited higher activity in tumor antigen presentation, immune cell priming and activation, T-cell recruitment, DC cell recruitment, monocyte recruitment, and immune cell infiltration into tumor tissue (Supplementary Figure S3F), which is in concordance with the findings from the immune cell infiltration analysis. Furthermore, we compared the activation levels of 13 immune functions among patients across various subtypes. The study revealed that the low NMBRS group displayed higher levels of immune functions, including APC_co_stimulation, HLA, T_cell_co−stimulation, and Type_II_IFN_Response (Figure 7I). By correlation analysis, we found a significant negative association of NMBRS with activated B cells, dendritic cells, and CD8 T cells and monocytes. Additionally, seven genes in NMBRS were highly correlated with tumor-infiltrating immune cells (Figures 7J, K).

### Immunotherapy-related analysis of NMBRS

**FIGURE 8:**
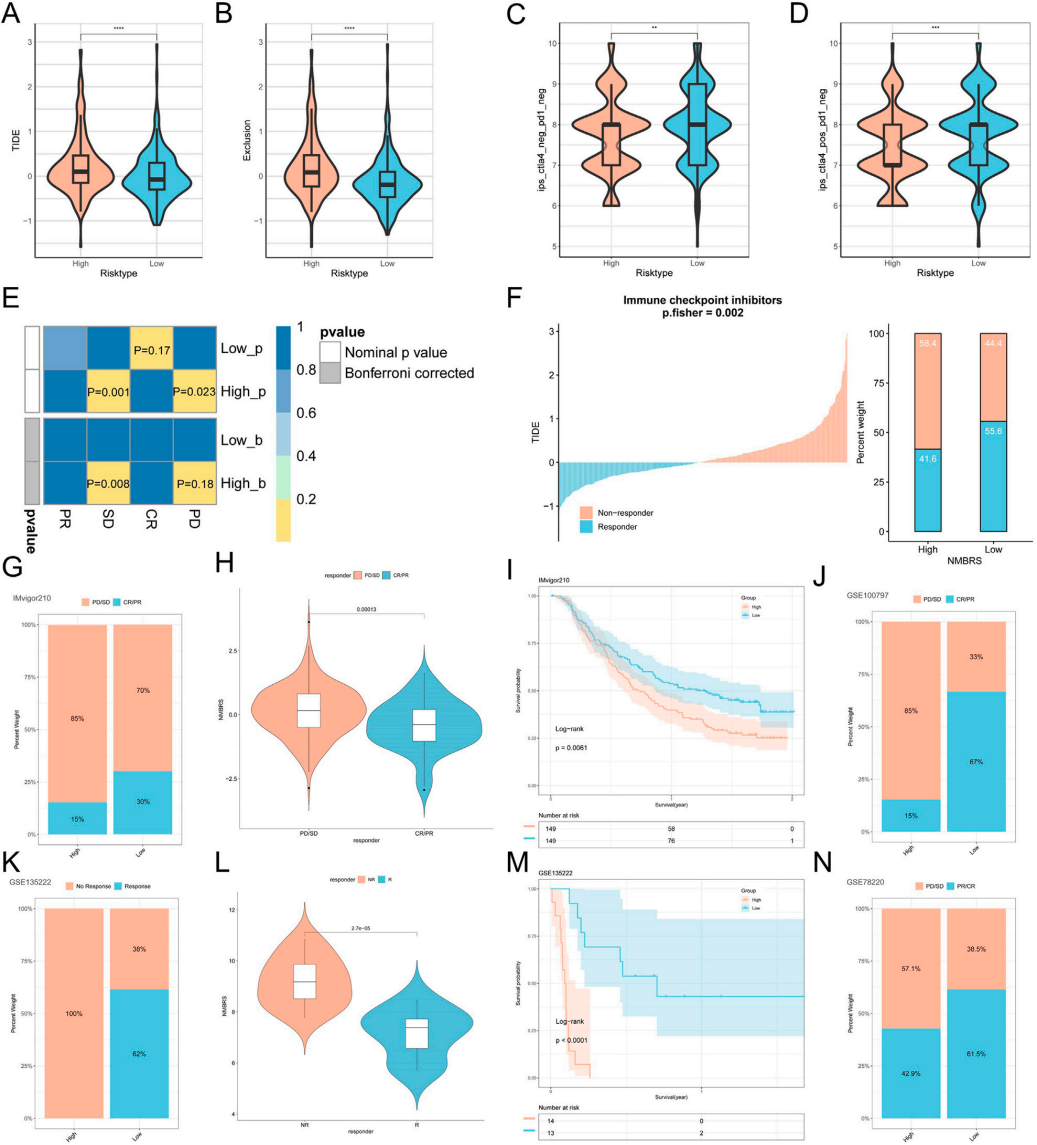
NMBRS is used to predict immunotherapy in LUAD patients. **(A, B)** TIDE score and T cell exclusion score differences in different subtypes of patients. **(C, D)** Differences in IPS scores among different subtypes of patients. **(E)** Submap analysis of different subtypes of patients. **(F)** Immunotherapy analysis results for LUAD patients from the TIDE database. **(G)** The percentage of CR/PR and PD/SD patients in the IMvigor210 cohort who received immunotherapy in the high NMBRS and low NMBRS groups. **(H)** Describe the Violin plot of the difference in NMBRS score for CR/PR and PD/SD patients from the IMvigor210 cohort. **(I)** Survival analysis for NMBRS from the IMvigor210 cohort. **(J)** The percentage with CR/PR and PD/SD in the GSE100797 cohort who received immunotherapy in both the high NMBRS and low NMBRS groups. **(K)** The percentage of patients in the GSE135222 cohort who responded or did not respond to immunotherapy in the high NMBRS and low NMBRS groups. **(L)** The Violin plots of the NMBRS score differences for responding and non-responding patients from the GSE135222 cohort. (M) Analysis of NMBRS survival from the GSE135222 cohort. **(N)** The percentage with CR/PR and PD/SD in the GSE78220 cohort who received immunotherapy in the high NMBRS and low NMBRS groups. (*P < 0.05; **P < 0.01; ***P < 0.001; ****P < 0.0001).

TIDE represents two core mechanisms simulating tumor immune evasion: T cell dysfunction and T cell exclusion computational methods (Jiang et al., 2018). We acquired and contrasted the TIDE scores for LUAD patients from the TIDE website and observed that the TIDE scores and T cell exclusion were notably higher in the high NMBRS group compared to the low NMBRS group (Figures 8A, B), indicating that tumor cells in the high NMBRS group are more likely to undergo immune escape. The IPS score is calculated using machine learning methods based on unbiased gene expression of representative cell types (Charoentong et al., 2017). We obtained and compared the IPS scores of LUAD patients from the TCIA database. The results showed significantly higher IPS scores in the low NMBRS group compared to the high NMBRS group (Figures 8C, D). This suggests that patients with low NMBRS scores may respond more favorably to immunotherapy. Next, we used the Submap algorithm to compare the similarity between different subtypes of patients and those receiving immunotherapy. We found that patients with higher NMBRS scores were more akin to non-responders to immunotherapy (PD, SD). In contrast, those with low NMBRS scores were more akin to responders to immunotherapy (CR) (Figure 8E). Based on the analysis of LUAD patients in the TIDE database, a higher proportion of patients in the high NMBRS group did not respond to immunotherapy (Figure 8F). To more accurately assess the immunotherapy response of different subtypes of patients, we collected four immunotherapy cohorts: IMvigor210, GSE78220, GSE135222, and GSE100797. In the IMvigor210 cohort, we observed a higher percentage of non-responding patients (PD, SD) in the high NMBRS group, as well as higher levels of NMBRS in patients with PD/SD, and a relatively poorer prognosis in patients with high NMBRS (Figures 8G–I). During the cohort study of GSE100797, we observed a higher percentage of PD/SD patients in the high NMBRS group (85%) and a higher proportion of CR/PR patients in the low NMBRS group (67%) (Figure 8J). In the cohort study in GSE135222, we noted a significantly higher percentage of non-responsive patients in the group with high NMBRS (100%), and the NMBRS levels of non-responsive patients were also higher, with the prognosis of high-risk patients being relatively poor (Figures 8K–M). In the GSE78220 cohort, we observed a higher percentage of PD/SD patients in the group with high NMBRS (57.1%), while the proportion of CR/PR patients was higher in the low NMBRS group (61.5%) (Figure 8N). The analysis of these results indicates that patients with low NMBRS scores are more inclined to benefit from immunotherapy than those with high NMBRS scores.

### Single-cell transcriptome analysis of NMBRS

**FIGURE 9:**
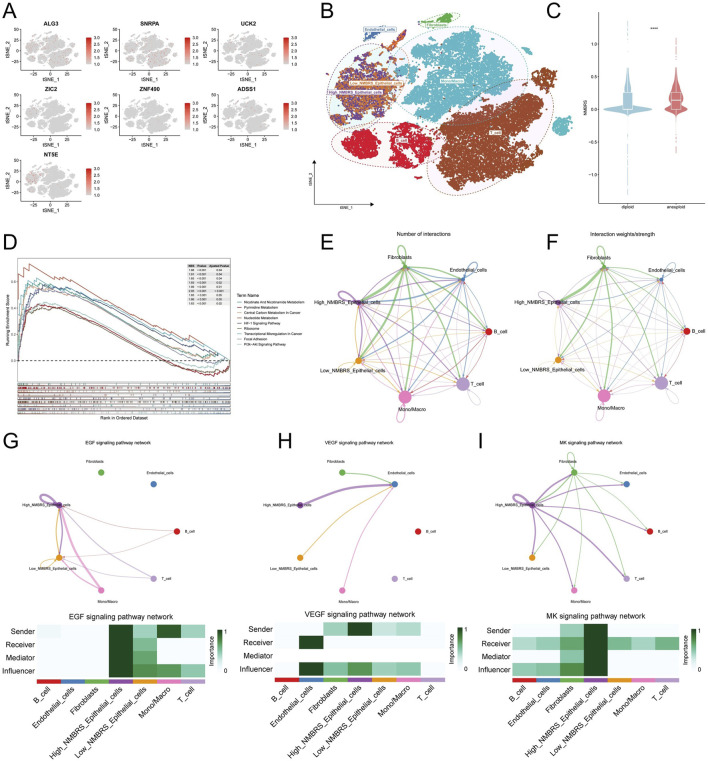
The correlation of NMBRS with single-cell characteristics. **(A)** Expression in various cell types of the 7 model genes. **(B)** Classification of epithelial cells into High_NMBRS_Epithelial_cells and Low_NMBRS_Epithelial_cells based on NMBRS. **(C)** Expression differences of NMBRS in malignant and normal cells. **(D)** Analysis for GSEA enrichment of differential genes between High_NMBRS_Epithelial_cells and Low_NMBRS_Epithelial_cells. **(E, F)** Cell communication relationships among a variety of cell types in TME. **(G–I)** Circo plots show EGF, VEGF, and MK signaling pathway networks, and Heatmap displays the effects of various cell types in these pathway networks. (*P < 0.05; **P < 0.01; ***P < 0.001; ****P < 0.0001).

First, we analyzed the expression of ALG3, SNRPA, UCK2, ZIC2, ZNF490, ADSS1 and NT5E at the single cell level (Figure 9A). The outcome indicated that these genes are predominantly expressed in epithelial cells, T cells, and Mono/Macro. Then, based on the expression of the modeling genes and their corresponding coefficients, we classified epithelial cells into two subgroups: High_NMBRS_Epithelial_cells and Low_NMBRS_Epithelial_cells (Figure 9B). We observed that the NMBRS levels in malignant epithelial cells were relatively high (Figure 9C). We differentially analyzed High_NMBRS_Epithelial_cells and Low_NMBRS_Epithelial_cells using the “FindMarkers” function and explored the pathways involved in these genes by GSEA enrichment analysis. The results suggest that these differential genes are primarily engaged in pathways that are closely tied to the formation and progression of tumors, such as Central Carbon Metabolism In Cancer, Nucleotide Metabolism, Hif−1 Signaling Pathway, Pi3k−Akt Signaling Pathway (Figure 9D). We subsequently employed the “Cellchat” R package to investigate the communication relationships between different cell types within the tumor microenvironment. Our research findings suggest that High_NMBRS_Epitheial_cells have a closer interaction with stromal cells and immune cells in the tumor microenvironment (Figures 9E, F) and play stronger regulators and influencers in EGF signaling, VEGF signaling, and MK signaling (Figures 9G–I). These signaling pathways are pivotal in tumor initiation and angiogenesis (Apte et al., 2019).

### Drug sensitivity analysis of different subtypes of patients

We utilized the R software package “oncoPredict” to estimate the sensitivities of LUAD patients to 198 chemotherapeutic drugs, comparing and identifying common chemotherapy-sensitive drugs for different patient subtypes. The results indicated that patients with high NMBRS scores showed greater sensitivity to chemotherapy drugs such as Paclitaxel, Lapatinib, Gefitinib, and 5-Fluorouracil (Supplementary Figures S4A–D). Patients with low NMBRS scores showed greater sensitivity to Doramapimod, Ribociclib, Nutlin-3a (−), and Oxaliplatin (Supplementary Figures S4E–H).

### Screening and pan-cancer analysis of key genes in nucleotide metabolism

**FIGURE 10:**
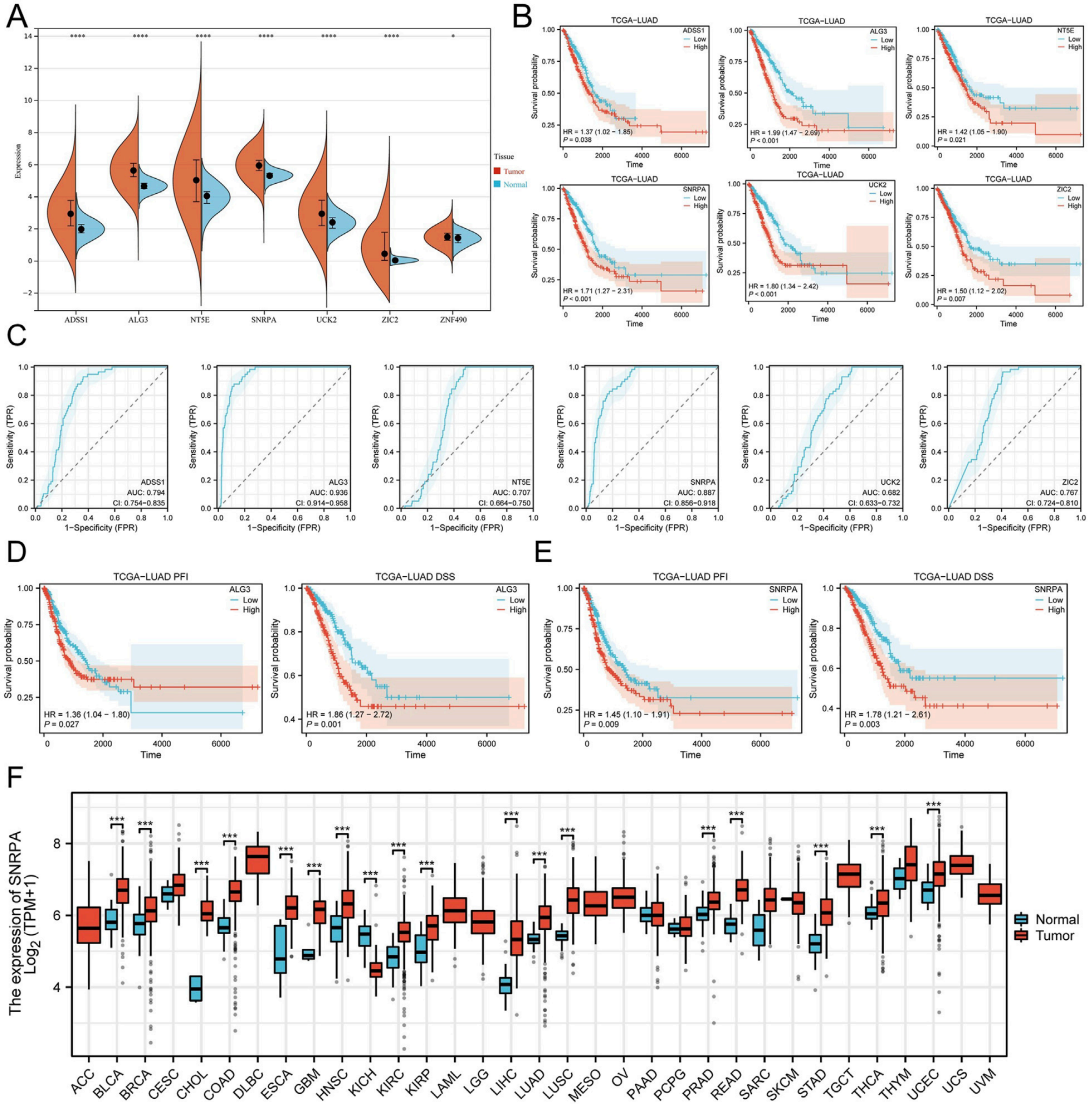
Screening of core genes and pan-cancer analysis. **(A)** Expression differences of the seven modeling genes in normal and tumor tissues. **(B)** Survival analysis of ADSS1, ALG3, SNRPA, NT5E, ZIC2, and ZNF490. **(C)** ROC diagnostic curves of ADSS1, ALG3, SNRPA, NT5E, ZIC2, and ZNF490. **(D)** Impact of ALG3 expression on DSS and PFI in LUAD patients. **(E)** Impact of SNRPA expression on DSS and PFI in LUAD patients. **(F)** Pan-cancer analysis of SNRPA expression. (*P < 0.05; **P < 0.01; ***P < 0.001; ****P < 0.0001).

First, we investigated the expression changes of seven model genes in normal and tumor tissues. It was found that in the TCGA cohort, ADSS1, ALG3, SNRPA, NT5E, UCK2, ZIC2, and ZNF490 were expressed at higher levels in tumors (Figure 10A). We also observed similar results in the GSE31210 and GSE116959 cohorts (Supplementary Figures S5A, B). Next, we examined the association of seven modeling genes with prognosis in LUAD patients, and the results indicated that ADSS1, ALG3, SNRPA, NT5E, and ZIC2 were remarkably correlated to the prognosis of LUAD patients (Figure 10B). ROC diagnostic curve analysis showed that ALG3 (AUC: 0.936) and SNRPA (AUC: 0.887) demonstrated relatively high accuracy in the diagnosis of LUAD (Figure 10C). Then, we explored the effects of ALG3 and SNRPA on the PFI and DSS of LUAD patients. The results indicated that the patients with high ALG3 and SNRPA expression performed poorly in PFI and DSS, with SNRPA having an enormous prognostic impact on LUAD patients (Figures 10D, E). Therefore, we chose SNRPA as the critical gene for nucleotide metabolism for further research. In the TCGA pan-cancer cohort study, we performed a thorough analysis of SNRPA expression, and the findings indicated that SNRPA is highly expressed across various tumor types (Figure 10F; Supplementary Figure S5C). According to pan-cancer COX analysis, SNRPA was identified as a risk factor for the prognosis of patients with various tumor types (Supplementary Figure S5D). To delve deeper into the clinical significance of SNRPA, we examined its correlation with the clinicopathological features of LUAD patients. The outcome indicated that SNRPA expression was notably elevated in males, late stages, M1 stages, and patients under the age of 65 (Supplementary Figure S5E). This suggests that SNRPA may be associated with the progression of LUAD.

### SNRPA stRNA-seq characterization in LUAD

To further explore the expression characteristics and role of SNRPA in lung adenocarcinoma. We explored different stages of LUAD (AIS, MIA, IAC). In adenocarcinoma *in situ* (AIS) subtype sections, SNRPA was widely expressed in the Cancer, Normal epithelium, Stromal, and Lymph regions (Supplementary Figures S6A, B). In microinvasive adenocarcinoma and invasive adenocarcinoma (MIA, IAC) subtype sections, SNRPA was more centrally expressed in the cancer region (Supplementary Figures S6C–F). These results suggest that SNRPA is widely expressed in cancer tissues and is associated with the progression of lung adenocarcinoma.

### Experimental validation of SNRPA’s role in LUAD progression

**FIGURE 11:**
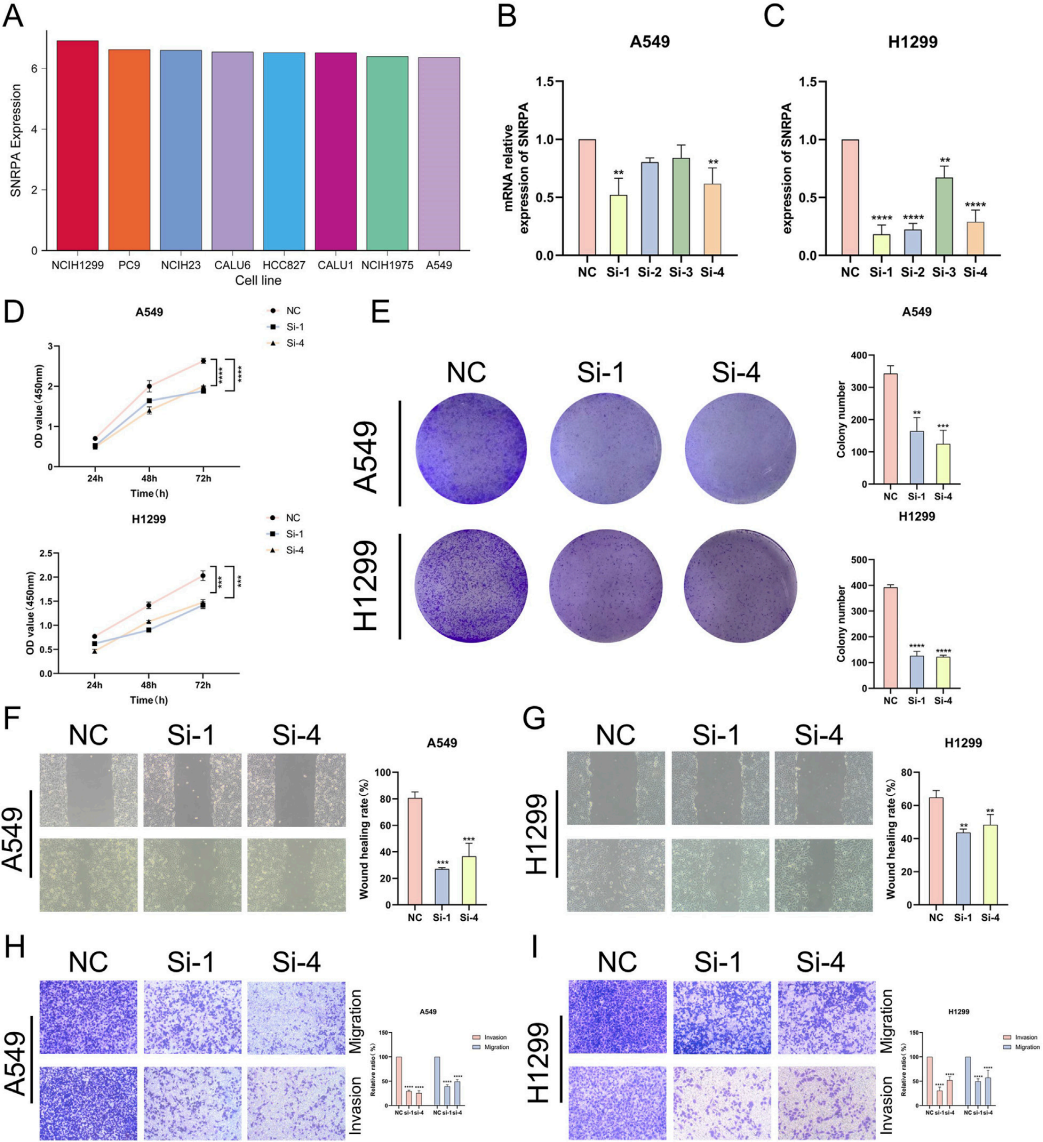
Experimental validation of SNRPA’s function. **(A)** mRNA expression levels for SNRPA in the LUAD cell line in the CEEL database. **(B, C)** RT-qPCR is used to detect the transfection efficiency for si-SNRPA into A549 and H1299 cell lines. **(D)** CCK-8 detects SNRPA, which influences the growth and proliferation of the A549 and H1299 cell lines. **(E)** Colony formation assay to detect SNRPA influences the proliferation in A549 and H1299 cell lines. **(F, G)** A wound healing assay was performed to detect SNRPA’s impact on the migration process of A549 and H1299 cell lines. **(H, I)** Transwell assay to validate SNRPA affects the migration and invasion of A549 and H1299 cell lines. (*P < 0.05; **P < 0.01; ***P < 0.001; ****P < 0.0001).

We acquired mRNA expression data for SNRPA from the CCLE database in the LUAD cell line and found that SNRPA is highly expressed in eight cell lines: NCIH1299, PC9, NCIH23, CALU6, HCC827, CALU1, NCIH1975, and A549 (Figure 11A). To explore the oncogenic role of SNRPA in LAUD, we designed siRNAs for SNRPA knockdown and transfected them into A549 and H1299 cells (Si1, Si2, Si3, Si4). RT-qPCR was used to evaluate the transfection efficiency, revealing that the relative expression levels of SNRPA were notably decreased following transfection with Si1 and Si4 (Figures 11B, C). To further verify the effect of SNRPA in proliferation, we conducted CCK-8 and colony formation assays. The CCK-8 assay outcome indicated that the proliferation capacity for A549 and H1299 cells in the knockdown groups (Si1, Si4) was notably decreased when compared to the control group (NC) (Figure 11D). Colony formation assays also demonstrated that the inhibition of SNRPA expression dramatically suppressed the growth of A549 and H1299 cells (Figure 11E). In addition, we conducted a wound healing assay, which indicated that the migratory ability of A549 and H1299 cells in the knockout group (Si1, Si4) was markedly attenuated (Figures 11F, G). Transwell assay results also indicated that knocking down SNRPA dramatically inhibited the migratory and invasive abilities of A549 and H1299 cells (Figures 11H, I). Together, these analyses suggest that SNRPA plays a critical function in the proliferation, migration, and invasion of lung adenocarcinoma and that disrupting the expression of SNRPA could effectively impede these processes in lung adenocarcinoma.

## Discussion

Lung adenocarcinoma is a highly malignant and heterogeneous tumor type (de Sousa and Carvalho, 2018), serving as the primary histological subtype of lung cancer, and poses a severe threat to people’s health and safety. Metabolic reprogramming, a fundamental characteristic of cancer, contributes to the provision of essential substrates and energy for tumor cell growth, proliferation, and metastasis. Pathways such as glucose, fatty acid, and amino acid metabolism have been demonstrated to play significant functions in the progression of precancerous lesions, tumor invasion and metastasis, and tumor resistance (Xia et al., 2021). In recent years, with the in-depth study of tumor metabolic reprogramming, the complex relationship between nucleotide metabolism and tumors has garnered attention (Doshi et al., 2023). As critical small molecules, nucleotides primarily function as building blocks for cellular nucleic acid synthesis and are pivotal in facilitating cell growth and proliferation (Rathbone et al., 1992). Increasing evidence indicates that nucleotide metabolism is vital for both the proliferative and non-proliferative processes of tumors (Zhao et al., 2024), as shown by Aarif et al.'s study, which found that inhibiting thymidylate synthase expression in breast cancer can effectively suppress EMT and reduces the ability of tumor cells to proliferate and migrate (Siddiqui et al., 2019). In addition, the effect that nucleotide metabolism has on tumor immunity should not be overlooked. ATP and ADP, as essential products of nucleotide metabolism, have been shown to function in immune stimulation and immunization processes (Zhang et al., 2018). During tumor formation, when metabolic stress or hypoxia is present, tumors and immune cells can synthesize adenosine (Leone and Emens, 2018). Adenosine receptors are present on almost all immune cell surfaces (Faas et al., 2017; Antonioli et al., 2019), and adenosine can inhibit anti-tumor immune responses by binding to adenosine receptors on dendritic cells and effector T cells (Panther et al., 2003). These studies indicate a significant association between nucleotide metabolism and tumor development, progression, anti-tumor immunity, and immunotherapy. However, there are relatively few reports on the relationship between nucleotide metabolism and prognosis, immunization and immunotherapy in LUAD.

By constructing a prognostic model, the present study reveals the prognostic and immunotherapeutic features of nucleotide metabolism in lung adenocarcinoma. We first elucidated the association between nucleotide metabolism and LUAD at the single-cell transcriptome scale. On the TCGA-LUAD queue, 51 nucleotide metabolism genes relevant to OS were determined by univariate COX regression analysis. Subsequently, variables were screened further, and the prognostic model (NMBRS) was structured using LASSO and Stepcox regression analyses. Survival time was markedly shorter in patients with high NMBRS than those with low NMBRS. Subsequently, the survival analysis results, timeROC curves, and model comparisons in the training and validation sets demonstrated the high accuracy and significant superiority of NMBRS for predicting the prognosis of LUAD patients. The genomic mutation analysis showed that genomic instability and mutation frequency were significantly higher in patients with high NMBRS scores. Functional enrichment analysis revealed marked enrichment in pathways relevant to nucleotide metabolism, cell cycle, and cell proliferation in high NMBRS patients. Pathways enriched in the high NMBRS group indicate their essential part in developing LUAD, suggesting a close connection between nucleotide metabolism and tumor pathways. Moreover, we found that the two subtypes of patients have different TME characteristics. Patients with low NMBRS exhibit high immune cell and stromal cell infiltration and expression of tumor immune-related molecules, tending more towards “hot tumor” characteristics. Patients with high NMBRS show low immune cell infiltration, T cell exclusion properties, and immune escape characteristics, leaning more towards “cold tumor” characteristics. Notably, NMBRS accurately predicted the immune therapy response in LUAD patients. In multiple external validation cohorts, patients with high NMBRS were significantly inclined to be non-responders to immune therapy, whereas patients with low NMBRS exhibited good immune therapy responses.

SNRPA is a protein composed of 282 amino acids with two RNA-binding domains. In conjunction with some neighboring amino acids, the RNA-binding domain at the N-terminus is crucial for its interaction with U1 small nuclear RNA (snRNA) (Price et al., 1998). The SNRPA protein, encoded by the SNRPA gene on chromosome 19q13.2 (Bai et al., 2013), is vital for forming the spliceosome and promoting mRNA splicing. It is involved in the SMN-dependent snRNP biosynthesis pathway, which regulates mRNA polyadenylation (Gunderson et al., 1998). Previous studies have shown that SNRPA promotes an EMT-like process in hepatocellular carcinoma cells by activating the NOTCH1/Snail pathway, thereby accelerating metastasis (Mo et al., 2023). In gastric cancer, the higher expression of SNRPA promotes gastric cancer cell development by activating NGF expression (Dou et al., 2018). In this study, we found that interfering with SNRPA expression could significantly inhibit the proliferation, migration, and invasion process of lung adenocarcinoma cells by *in vitro* cellular experiments. This suggests that SNRPA plays an essential role in lung adenocarcinoma occurrence and development.

In summary, this study constructed a prognostic signature related to nucleotide metabolism in lung adenocarcinoma. This signature showed high accuracy and superiority in predicting the prognosis of lung adenocarcinoma patients and can be used as a potential guideline for lung adenocarcinoma immunotherapy, clinical drug selection. In addition, *in vitro* cellular experiments demonstrated that SNRPA plays an essential role in the occurrence and development of LUAD and can serve as a potential biomarker for lung adenocarcinoma. However, our research also has restrictions and shortcomings. Firstly, the test and validation set data utilized in our analysis are from publicly available databases, and the inherent sample selection criteria may affect the results of the model construction to a certain extent. Secondly, the limited sample size of this study requires a larger cohort and sample size to validate our findings further. Finally, the present study validated SNRPA’s role in LUAD proliferation, migration, and invasion only by *in vitro* cell line function experiments, lacking an investigation into the potential molecular biological mechanisms by which SNRPA functions in LUAD. In future research, we intend to investigate the precise mechanisms through which SNRPA influences the progression of LUAD, employing *in vivo* and *in vitro* experimental approaches and exploring novel anticancer drugs that target SNRPA for action.

## Conclusion

In this study, we constructed a nucleotide metabolism-related prognostic risk model based on nucleotide metabolism-related genes. This prognostic signature can accurately predict the prognosis of LUAD patients and provide potential guidance for immunotherapy. In addition, we found that nucleotide metabolism-associated LUAD subtypes differed significantly in various aspects, such as genomic mutations, functional enrichment, and tumor immune characteristics. Finally, the nucleotide metabolism-related gene SNRPA in the model was shown to have an essential role in promoting LUAD onset and malignant progression.

## Data Availability

The original contributions presented in the study are included in the article/Supplementary Material, further inquiries can be directed to the corresponding author.

## Abbreviations:

LUAD, Lung adenocarcinoma; TCGA, The cancer genome atlas; GEO, Gene expression omnibus; NMBRS, Nucleotide metabolism-related prognostic risk score; TME, Tumor microenvironment; Lasso, Least absolute shrinkage and selection operator; OS, Overall survival; DSS, Disease specific survival; PFI, Progress free interval; GO, Gene ontology; KEGG, Kyoto encyclopedia of genes and genomes; ROC, Receiver operating characteristic; DCA, Decision curve analysis; TMB, Tumor mutation burden; MATH, Mutant-allele tumor heterogeneity; CNV, Copy number variations; SNV, Single nucleotide variants; ssGSEA, Single sample gene set enrichment analysis.
