# Supplementary material for: Integration of single-cell transcriptomics and bulk transcriptomics to explore prognostic and immunotherapeutic characteristics of nucleotide metabolism in lung adenocarcinoma
Source: Front Genet. 2025 Jan 8;15:1466249. doi: 10.3389/fgene.2024.1466249 (PMC11750784; doi:10.3389/fgene.2024.1466249)
Supplement: Supplementary file 2 [file Image1.pdf]

Supplementary Figure

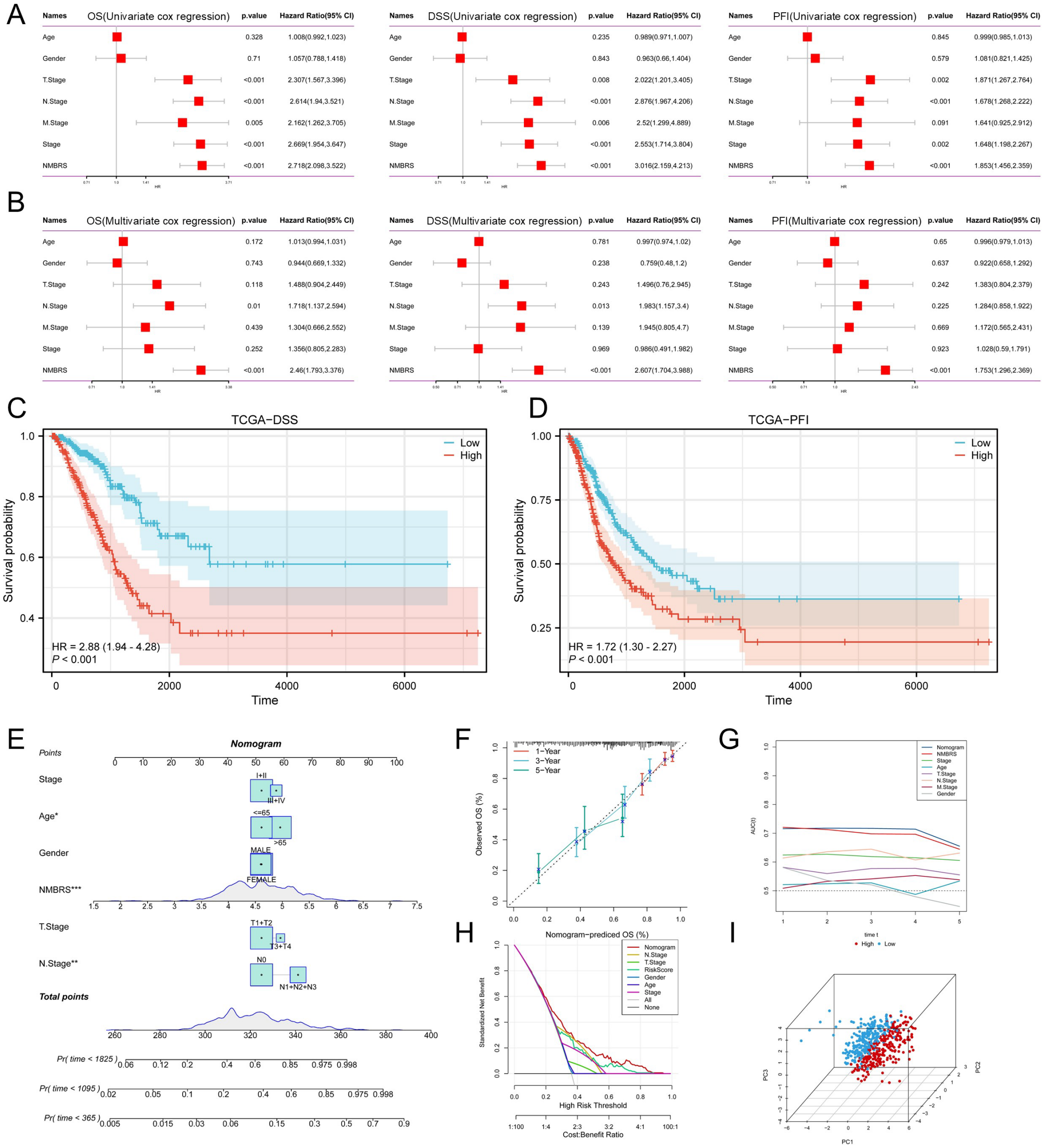

Supplementary Figure 1. Independent prognostic analysis and construction of a nomogram

(A) Univariate COX regression analysis of NMBRS and various clinicopathological factors in the TCGA-LUAD cohorts for OS, DSS, and PFI. (B) Multivariate COX regression analysis for NMBRS in the TCGA-LUAD cohort and various clinicopathologic factors on OS, DSS, and PFI. (C-D) Predictive performance of NMBRS in DSS and PFI. (E) Construction of the nomogram based on NMBRS. (F) Nomogram calibration curves for 1-year, 3-year, and 5-year projections. (G) timeROC curves of the nomogram model. (H) The DCA curves for nomograms, NMBRS, and other clinicopathologic factors. (I) Results for PCA analysis of NMBRS.

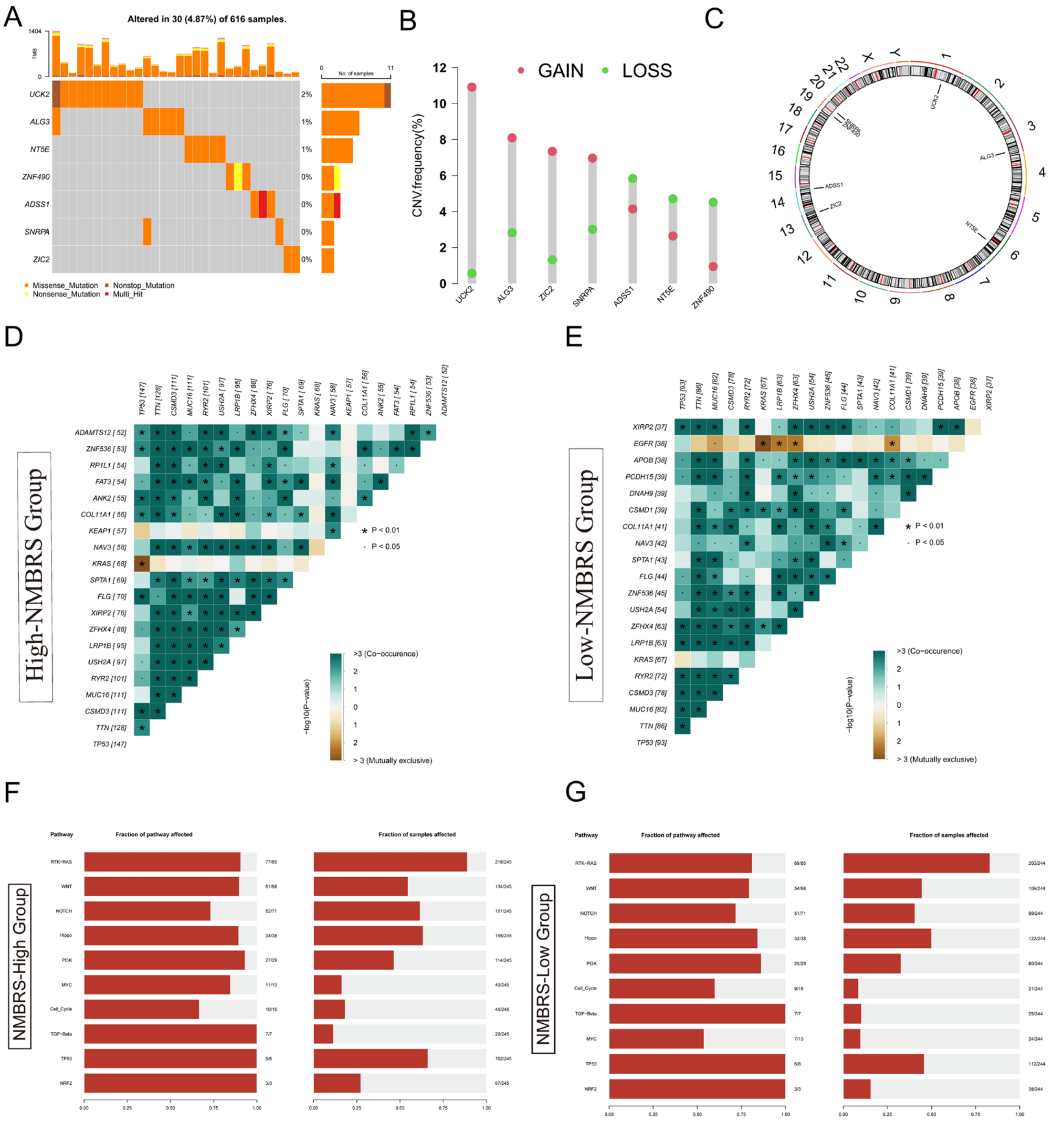

Supplementary Figure 2. Mutation characteristics of NMBRS

(A) Waterfall plot of SNV mutations in 7 modeling genes. (B) CNV mutation status of 7 modeling genes. (C) Distribution of 7 modeling genes on chromosomes. (D) Interaction network of the top 20 mutated genes in the high NMBRS group. (E) Interaction network of the top 20 mutated genes in the low NMBRS group. (F) Cancer-related pathways affected by mutated genes in the high NMBRS group. (G) Cancer-related pathways affected by mutated genes in the low NMBRS group.

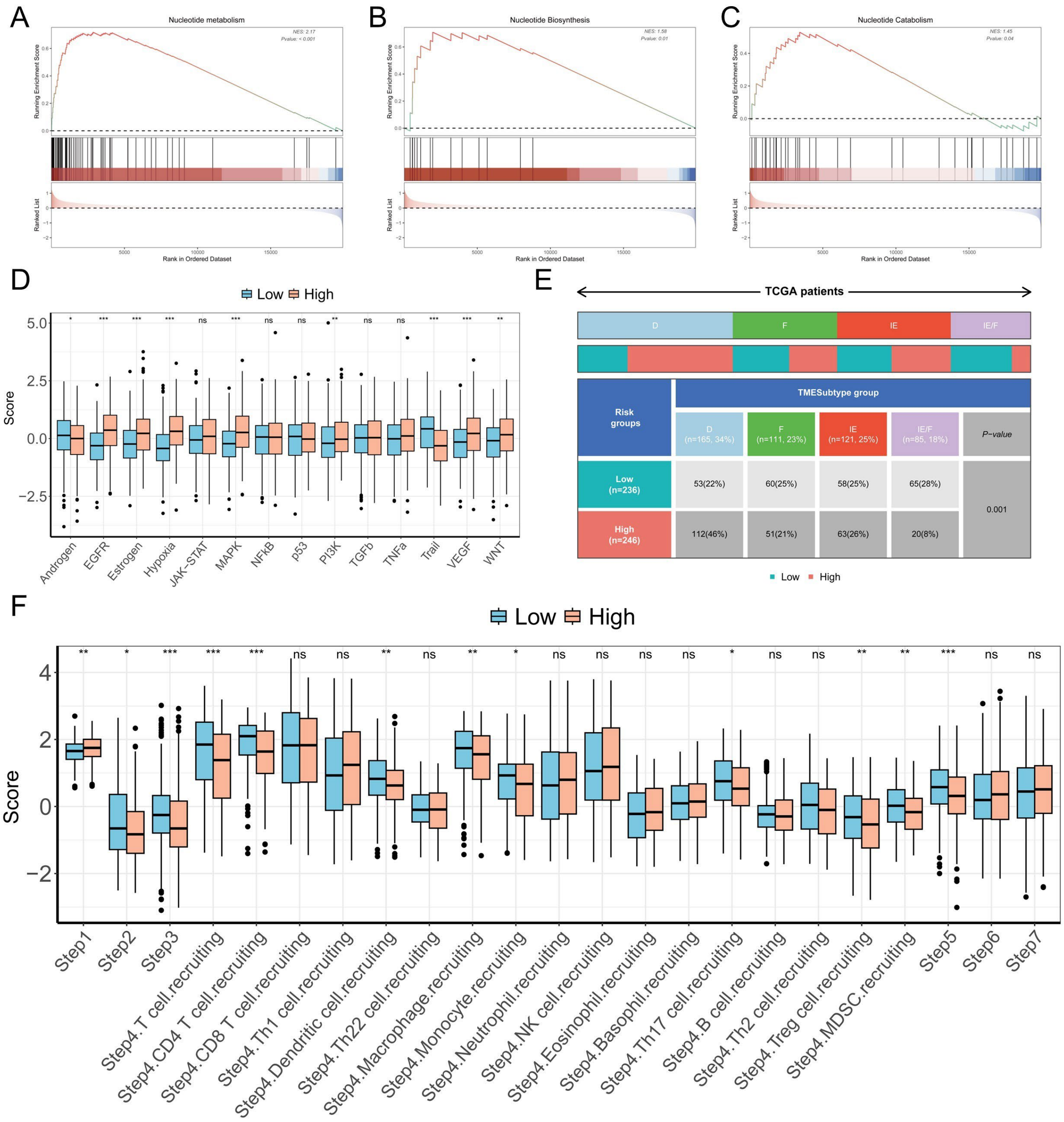

Supplementary Figure 3. Enrichment and immune characteristics of NMBRS function

(A-C) Enrichment of nucleotide metabolism-related pathways in patients of different subtypes. (D) Activation level of tumor signaling pathways in patients of different subtypes. (E) Distribution of TME subtypes in patients with high and low NMBRS. (F) Activation levels of various steps in the immune seven-step cycle in patients of different subtypes. (*P < 0.05; **P < 0.01; ***P < 0.001; ****P < 0.0001).

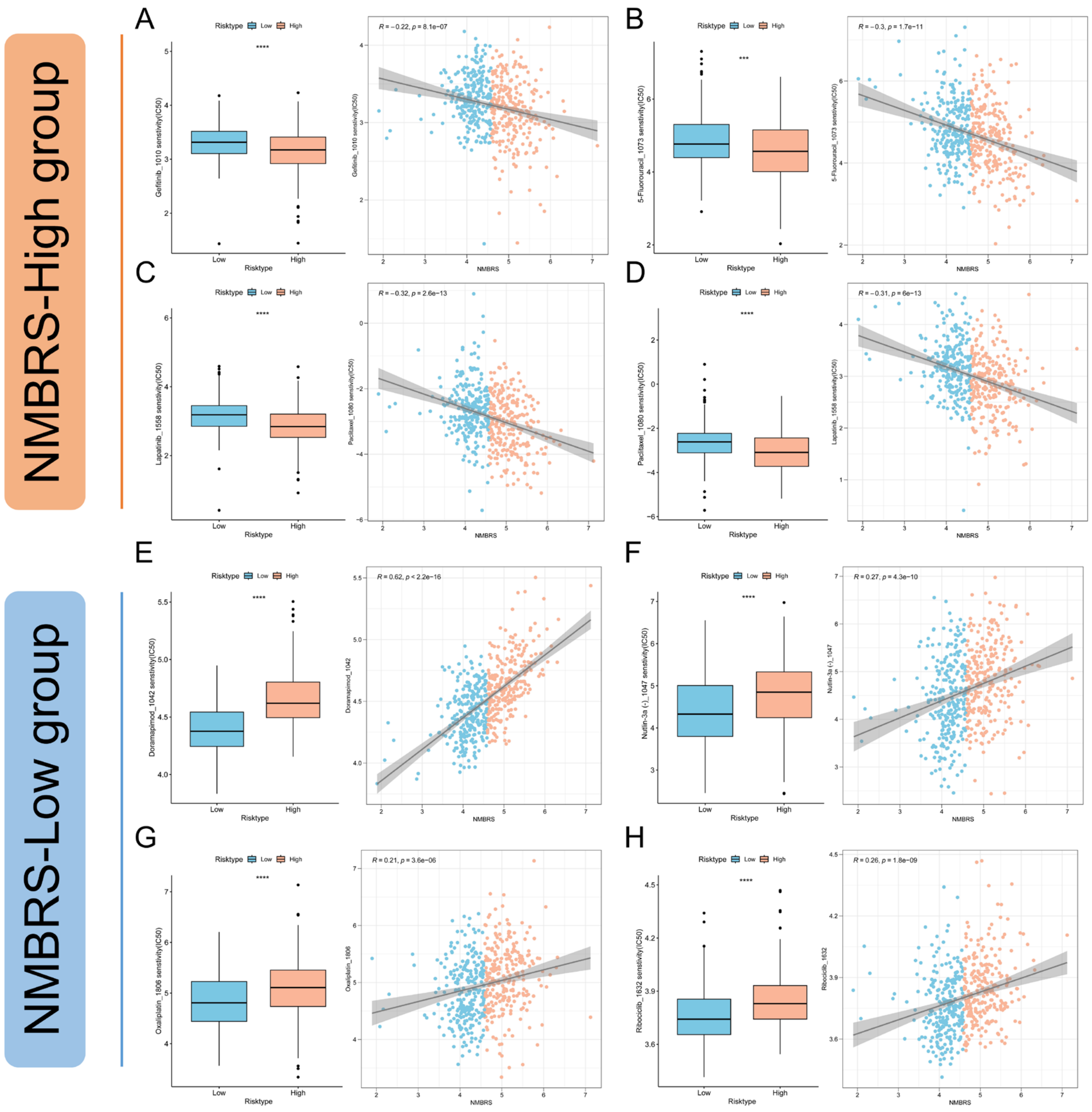

Supplementary Figure 4. Sensitivity analysis of chemotherapy drugs in patients with different subtypes

(A-D) Box plots showing the sensitivity of high NMBRS group patients and low NMBRS group patients to Gefitinib, 5-Fluorouracil, Lapatinib, and Paclitaxel. Correlation analysis indicates the correlation between NMBRS and IC50 of Gefitinib, 5-Fluorouracil, Lapatinib, and Paclitaxel. (E-H) Box plots showing the sensitivity of high NMBRS group patients and low NMBRS group patients to Doramapimod, Nutlin-3a(-), Oxaliplatin, and Ribociclib. Correlation analysis indicates the correlation between NMBRS and IC50 of Doramapimod, Nutlin-3a(-), Oxaliplatin, and Ribociclib. (*P < 0.05; **P < 0.01; ***P < 0.001; ****P < 0.0001).

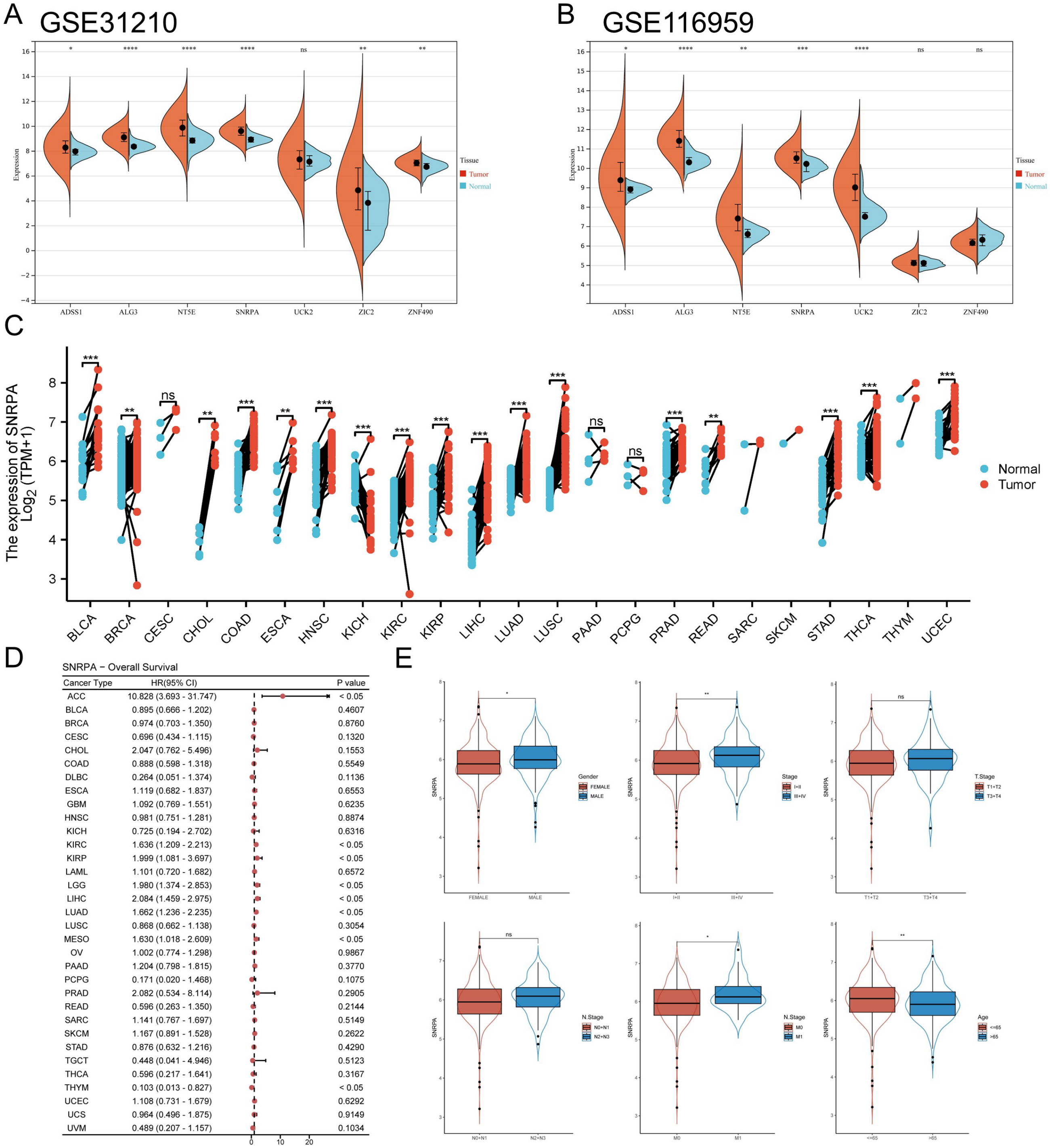

Supplementary Figure 5. Pan-cancer analysis of SNRPA

(A) Expression differences of the 7 modeling genes in the GSE31210 cohort. (B) Expression differences of the 7 modeling genes in the GSE116959 cohort. (C) Pan-cancer paired differential expression analysis of SNRPA. (D) Pan-cancer COX regression analysis of SNRPA. (E) Clinical pathological feature analysis of SNRPA. (*P < 0.05; **P < 0.01; ***P < 0.001; ****P < 0.0001).

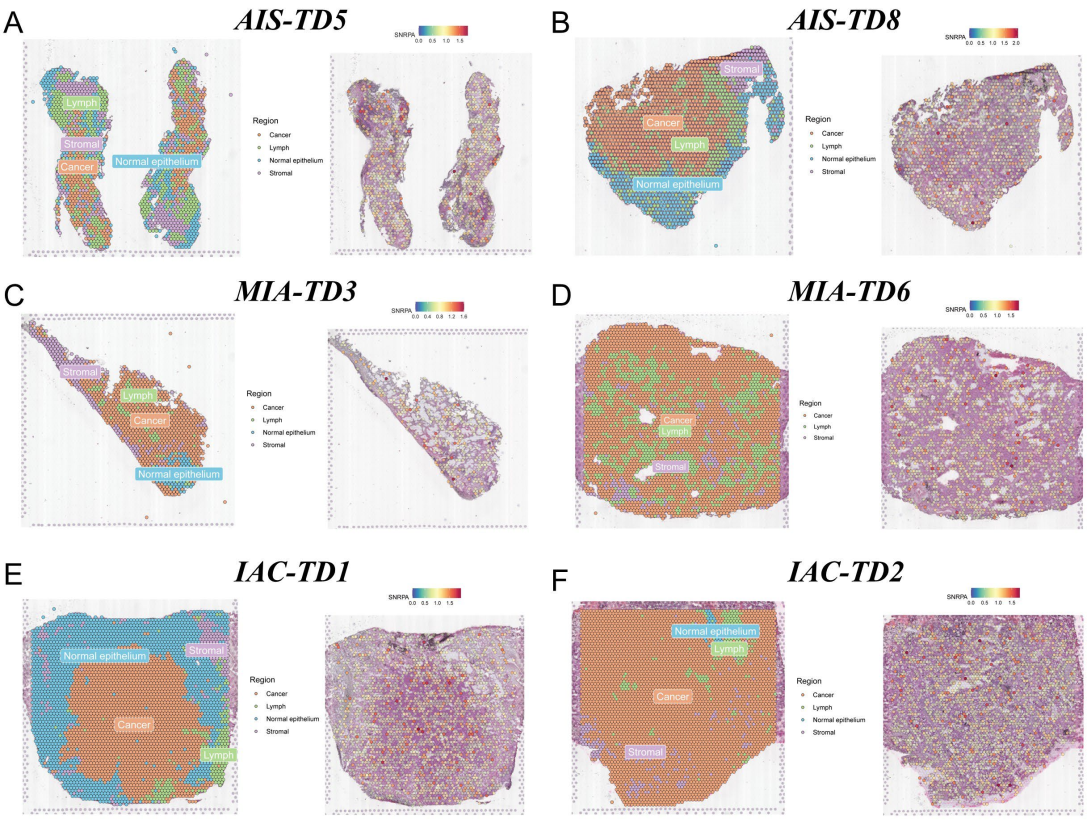

Supplementary Figure 6. Spatial transcriptomic analysis of SNRPA

(A-B) Spatial regions of AIS subtypes and spatial distribution of SNRPA in lung adenocarcinoma. (C-D) Spatial regions of lung adenocarcinoma MIA subtypes and spatial distribution of SNRPA. (E-F) Spatial regions of lung adenocarcinoma IAC subtypes and spatial distribution of SNRPA.
